# Microbial-Transferred Metabolites and Improvement of Biological Activities of Green Tea Catechins by Human Gut Microbiota

**DOI:** 10.3390/foods13050792

**Published:** 2024-03-04

**Authors:** You Su, Kaiyin Hu, Daxiang Li, Huimin Guo, Li Sun, Zhongwen Xie

**Affiliations:** 1 The College of Pharmacy, Anhui University of Chinese Medicine, Hefei 230012, China; suyoua-hu@163.com (Y.S.); kaiyinhuaht@163.com (K.H.); 2 State Key Laboratory of Tea Plant Biology and Utilization, School of Tea and Food Sciences and Technology, Anhui Agricultural University, Hefei 230036, China; dxli@ahau.edu.cn (D.L.); huiminguo@ahau.edu.cn (H.G.); 3 Center for Biotechnology, Anhui Agricultural University, Hefei 230036, China

**Keywords:** green tea catechins, human gut microbiota, metabolites, UHPLC-Q-Orbitrap-MS/MS, bioactivities, in vitro fermentation

## Abstract

Green tea catechins (GTCs) are dietary polyphenols with broad bioactivities that undergo extensive microbial metabolism in the human gut. However, microbial-transferred metabolites and their health benefits are not fully understood. Herein, the microbial metabolism of GTCs by human fecal microbiota and dynamic alteration of the microbiota were integrally investigated via in vitro anaerobic fermentation. The results showed that the human gut microbiota exhibited a strong metabolic effect on GTCs via UHPLC-MS/MS analysis. A total of 35 microbial-transferred metabolites were identified, far more than were identified in previous studies. Among them, five metabolites, namely EGCG quinone, EGC quinone, ECG quinone, EC quinone, and mono-oxygenated EGCG, were identified for the first time in fermented GTCs with the human gut microbiota. Consequently, corresponding metabolic pathways were proposed. Notably, the antioxidant, α-amylase, and α-glucosidase inhibitory activities of the GTCs sample increased after fermentation compared to those of the initial unfermented sample. The results of the 16S rRNA gene sequence analysis showed that the GTCs significantly altered gut microbial diversity and enriched the abundancy of *Eubacterium*, *Flavonifractor*, etc., which may be further involved in the metabolisms of GTCs. Thus, these findings contribute to a better understanding of the interactions between GTCs and gut microbiota, as well as the health benefits of green tea consumption.

## 1. Introduction

Green tea, made from the leaves and buds of *Camellia sinensis*, is one of the world’s most popular beverages [1]. The consumption of green tea has multiple health benefits, such as preventing metabolic syndrome and diabetes and reducing the risk of cardiovascular diseases and cancer [2,3,4,5]. These health-promoting effects can generally be attributed to the phenolic compounds in green tea, particularly catechins [6]. The four most abundant green tea catechins (GTCs) are (−)- epigallocatechin-3-gallate (EGCG), (−)- epigallocatechin (EGC), (−)- epicatechin-3-gallate (ECG), and (−)- epicatechin (EC) [7]. GTCs are characterized by a 3,5-dihydroxyphenyl A-ring, a di- or tri-hydroxyphenyl B-ring and a dihydropyran C-ring, which is fused to the A-ring. Additionally, the C-ring contains a hydroxyl or galloyl group at the C3 position (Figure S1) [8]. In recent years, GTCs have received significant attention due to their antioxidant, antibacterial, anti-inflammatory, and anti-cancer properties [2,7]. However, only a small fraction of consumed GTCs can be digested and absorbed. GTCs are considered to be poorly bioavailable in the small intestine [9]. The relatively low bioavailability of GTCs contrasts with their beneficial effects on human health [10]. Most GTCs reach the colon, where they are extensively metabolized by colonic microbiota into smaller compounds [11]. Therefore, the bioactivities of catechins largely depend on their biotransformation by the gut microbiota. However, how and what GTC metabolites are biotransformed by the human gut microbiota are largely unknown.

The gut microbiota is a resident microbial community present in the gastrointestinal tract composed of 10^13^–10^14^ commensal [12], symbiotic, and opportunistic pathogenic microorganisms. The collective metagenome of gut microbiota, referred to as the microbiome, includes many metabolic genes not present in the human genome, making the colon a bioreactor that has great metabolic potential [13].Growing evidence suggests that the gut microbiota is key to modulating the bioavailability and biological activities of dietary polyphenols [14]. For example, a study was reported that the biotransformation of Ziziphi Spinosae Folium polyphenol compounds by the human gut microbiota exhibited higher antioxidant activity than the original polyphenols [15]. Thus, studying biotransformation carried out by the human gut microbiota is essential for understanding the actual health-promoting effects of GTCs. Additionally, the homeostasis of the gut microbiota contributes to the prevention of many diseases [16,17]. Hence, the gut microbiota’s composition can affect the host’s health. Several studies have shown that green tea promotes a more health-beneficial mouse gut microbiota composition [9,17]. Nevertheless, to date, few reports on the influence of pure GTCs and their metabolites on human gut microbiota composition exist.

This study combines UHPLC-Q-Orbitrap-MS/MS and Compound Discoverer 2.1 software to screen and identify microbial metabolites of GTCs through in vitro anaerobic fermentation carried out by the human gut microbiota. A total of 35 microbial-transferred metabolites were identified. Among them, five metabolites were reported for the first time. Then, alterations in the antioxidant, α-amylase, and α-glucosidase inhibitory activities of GTCs at different fermentation times were investigated. The microbiota’s dynamics during the fermentation process were also evaluated using full-length 16S rRNA sequencing. The results obtained during this study will further our understanding of the microbial biotransformation of GTCs within the human gut and provide insights into the important influence of microbial metabolites on the overall bioactivities of GTCs.

## 2. Materials and Methods

### 2.1. Chemicals and Reagents

The standard substances EGCG, EGC, ECG, and EC, all with a purity of 98%, were purchased from Chengdu Munster Biotechnology Co., Ltd. (Chengdu, China). Gallic acid, Pyrogallol, 4-phenylbutyric acid, 3-phenylpropionic acid, phenylacetic acid, 3-(3′,4′-dihydroxyphenyl)propanoic acid, 2-(4′-hydroxyphenyl)acetic acid, 2-(3′,4′-dihydroxyphenyl)acetic acid, and 4-hydroxybenzoic acid, all with purities of at least 98%, were obtained from Yuanye Bio-Technology Co., Ltd. (Shanghai, China). 2,2′-azino-bis-(3-ethylbenzothiazoline-6-sulfonic acid) (ABTS), 2,2-diphenyl-1-picrylhydrazyl (DPPH), α-glucosidase, α-amylase, 3,5-dinitrosalicylic acid (DNS), and P-nitrophenyl-α-D-glucopyranoside (pNPG) were also purchased from Yuanye Bio-Technology Co., Ltd. (Shanghai, China).

The mass spectrometer (MS)-grade methanol, acetonitrile, and formic acid of the guaranteed reagent were purchased from Thermo Fisher Scientific Co. (Waltham, MA, USA). Deionized water (>18.2 MΩ/cm) was prepared using distilled water processed via a Milli-Q water purification system (Millipore, Billerica, MA, USA). Other reagents were of analytical grade.

### 2.2. Sample Preparation

In this study, GTCs containing four catechins monomers (EGCG, EGC, ECG, EC), the content percentages of which were 52.00% (EGCG), 20.85% (EGC), 20.85% (ECG), and 6.30% (EC), respectively, were used to mimic those found in the typical Chinese green tea Huangshan Mao Feng, using the data obtained in prior experiments [18]. The GTCs sample was diluted in sterile ultrapure water before being used for in vitro fermentation, resulting in a concentration of 0.1 mg/mL.

### 2.3. Fecal Sample Collection and Biotransformation of GTCs by Human Gut Microbiota

The in vitro fecal fermentation of GTCs was performed based on the methodology described in a previous publication [19], albeit with some modifications. Fresh human feces samples were collected from eight healthy Chinese volunteers, including four males and four females, 18 to 24-year-old with BMI between 18.5 and 23.5, who reported that they do not smoke and had no consumption of tea in the two weeks prior to the donation, as well as not currently taking medication and with no use of antibiotics in the last three months. More information about the volunteers can be found in Table S2. First, the 1 g faces samples collected from each of the eight healthy volunteers were immediately mixed, collected into sterile vials, homogenized with sterilized DPBS (0.1 M, pH 7.2) to yield a 10% (*w*/*v*) suspension, and filtered through four layers of sterile gauze sponges. Next, the filtered suspension (2.7 mL) was added to a general anaerobic medium (GAM) broth (24.3 mL) and incubated at 37 °C in the anaerobic chamber (5% H_2_, 10% CO_2_, and 85% N_2_) for 12 h to activate the bacteria. Subsequently, 27 mL of HFS was mixed with 3 mL of GTCs in water, reaching a final GTCs concentration of 0.1 mg/mL. As a control, 3 mL of water was added to 27 mL of HFS. The mixtures were then incubated at 37 °C in the anaerobic chamber for 48 h. After 0, 2, 4, 8, 12, 24, and 48 h of fermentation, 1 mL samples were taken and mixed with 3 mL of pre-cold acetonitrile to stop the fermentation. After centrifugation (30 min, 12,000 rpm, 4 °C), the supernatants were collected to analyze the metabolites via UPLC-MS/MS. For the gut microbiota composition analysis, 3 mL samples were collected after fermentation times of 0, 12, 24, and 48 h and immediately frozen at −80 °C until bacterial DNA extraction was performed. All the experiments were repeated in triplicate.

### 2.4. Analysis of the Microbial Metabolites of GTCs via UHPLC-Q-Orbitrap-MS/MS

To identify the microbial metabolites of GTCs, the obtained supernatants were analyzed using the UHPLC-Q-Orbitrap-MS/MS system (Thermo Fisher Scientific, Waltham, MA, USA) equipped with a binary pump solvent management system. The protocol was slightly modified from that used in the previous literature [20]. In brief, a small volume of 2.0 μL sample was separated using an Acquity UHPLC BEH C18 column (150 mm × 2.1 mm, 1.8 µm; Waters, Milford, MA, USA), the temperature of which was maintained at 40 °C. The A (0.75% formic acid in water) and B (0.75% formic acid in CAN) solutions were used as the mobile phase. The flow rate was set at 0.3 mL/min under the following gradient elution conditions: 0–2 min, 99% A; 2–22 min, 99-1% A; 22–25 min, 1% A. The mobile phase was adjusted to fit the starting conditions for 1 min, followed by equilibration for 4 min.

For mass spectrometric analysis, a Thermo Q Exactive Focus Oribitrap high-resolution mass spectrometer (Thermo Fisher Scientific, Bremen, Germany) supplied with a heated electro-sprayer for ionization (HESI) was coupled to the UHPLC system. Nitrogen was used as both the sheath gas (15 arbitrary units) and auxiliary gas (10 arbitrary units). The mass spectrometer was operated in both negative and positive modes. The ESI parameters were based on the methodology described in a previous paper [21]. Data acquisition and reprocessing were performed using Xcalibur (version 3.0, Thermo Fisher Scientific, Waltham, MA, USA).

### 2.5. Analytical Strategy Based on UHPLC-Orbitrap MS/MS

The cleavage patterns of flavonoids present in the published literature were adopted to systematically identify the metabolites of GTCs transferred by the human gut microbiota based on UHPLC-Q-TOF-MS/MS combined with online data acquisition and multifarious processing methods [22,23,24]. The analytical strategy’s flowchart is shown in Figure S2. (1) Data acquisition included online full-scan data acquisition using a combination of the full-scan and dd MS2 modes, yielding accurate MS and MS2 spectra. Metabolite screening and identification were performed using Compound Discoverer 2.1 software in conjunction with multiple postprocessing techniques. (2) Data analysis combined mass defect filters (MDF) with dynamic background subtraction (DBS) to screen metabolites and eliminate many interfering ions. High-resolution extracted ion chromatograms (HREIC) were then used to predict the metabolite molecular weights and formulas based on the accurate MS data. Diagnostic product ions (DPI) and neutral loss fragments (NLF) were utilized to identify potential GTC metabolites, the cleavage pathways of which resembled those of the four catechins. (3) Metabolite structure inference was based on accurate molecular weights and formulas, as well as cleavage patterns and related flavonoid compound biotransformation information, for the four catechins. (4) The Clog *p* values predicted using the ChemDraw 14.0 software distinguished between metabolite isomers, as metabolites with larger Clog *p* values generally eluted more slowly in reverse-phase chromatographic systems. (5) Finally, the inferred metabolite structures were further validated by comparing the retention times (RT) and MS2 data with those of standard compounds, as well as by reviewing research results related to catechins and structurally similar polyphenols present in the relevant literature.

### 2.6. Antioxidant Assay

DPPH radical scavenging activity was assessed via the previously described method, albeit with some modifications [19]. In brief, 50 μL of the diluted sample solution was added to 150 μL of the DPPH assay working solution (0.20 mM) in a 96-well microplate and incubated in the dark at room temperature for 30 min before we measured the absorbance at 517 nm. Then, the 70% (*v*/*v*) CAN solution was used, rather than the sample, as a negative control, and the DPPH scavenging rate was calculated.

The ABTS radical scavenging activity was measured through a previously described method [19]. A total of 20 μL of each sample at various concentrations was mixed with 180 μL of the ABTS working solution in a 96-well plate, and the absorbance was then measured at 734 nm after 6 min of incubation in the dark at 37 °C. The ABTS% was then calculated using an equation provided by the manufacturer.

### 2.7. Measurement of α-Amylase and α-Glucosidase Inhibitory Activity

Inhibitory assays for α-amylase and α-glucosidase were conducted based on previously reported method, albeit with slight modifications [25,26]. In brief, a mixture including 100 μL of each time point’s GTCs fermentation sample and 100 μL of a 1:1 U/mL enzyme solution in PBS (pH 6.8) was incubated at 37 °C for 15 min. Then, 50 μL of 1% soluble starch solution in PBS was added to the mixtures and incubated at 37 °C for 10 min. Finally, the DNS reagent was added to the mixture, which was further heated to 100 °C for 10 min. The absorbance was determined at a 540 nm wavelength using a microplate reader (SpectraMax i3x, Molecular Devices, San Jose, CA, USA). The α-amylase activity was calculated based on a method outlined in a previous publication. For the α-glucosidase activity inhibitory assay, a mixture including 40 μL of each time point’s fermentation sample was extracted, and 80 μL of enzyme solution (1 U/mL in PBS, pH 6.8) was incubated in a 96-well plate at 37 °C for 10 min. Before the absorbance was determined at a 405 nm wavelength using a microplate reader (SpectraMax i3x, Molecular Devices, San Jose, CA, USA), pNPG was added to the 96-well plate and incubated at 37 °C for 5 min.

### 2.8. Analysis of Gut Microbiota Composition

The gut microbiota in the fermentation solution was collected via centrifugation at 10,000× *g* for 5 min at 4 °C. The genomic DNA was extracted from each sample using the QiAamp Fast D.N.A. Stool Mini Kit (Qiagen, Hilden, Germany) according to the manufacturer’s instructions. The microbial community composition was analyzed through full-length 16S rRNA gene amplicon sequencing by Novogene, Beijing, China. The full V1–V9 region of the bacterial 16S rRNA gene was amplified using the universal primer sets 27 F (5′-AGAGTTTGATCCTGGCTCAG-3′) and 1492R (5′-GNTACCTTGTTACGACTT-3′). The PCR products were purified in identical quantities and sequenced using the PacBio platform. Lima was used to distinguish between the data in each sample based on the barcode sequence. SSR filtering was carried out, and Cutadapt software was used to remove the primers. After quality filtering was performed, the raw reads were clustered into operational taxonomic units (OTUs) with 97% sequence similarity using UPARSE. The representative sequence for each species’ OTU was then annotated against the Silva S.S.U. rRNA database with Mothur. The relative abundance of each OUT across all samples was calculated and used to perform further data mining.

### 2.9. Data Analysis

Raw UHPLC-MS/MS data were acquired using Thermo Xcalibur software (Version 3.0) and processed using Thermo CD software (Version 2.1). The microbial metabolites of GTCs by human gut microbiota were analyzed by Compound Discoverer 2.1 software. Data are expressed as the mean ± standard deviation (SD) and evaluated via Student’s t-test, and a value of *p* < 0.05 was considered to be statistically significant. The quantitative results were illustrated using GraphPad Prism software (Version 7.00).

## 3. Results

### 3.1. Identification of the Metabolites of GTCs Fermented via Human Fecal Fermentation In Vitro

The metabolism profiles of the GTCs during the 48 h of fermentation with the human gut microbiota were monitored via the established method of UHPLC-Q-Orbitrap-MS/MS data analysis, which constituted data-dependent acquisition (DDA). A total of 35 potential GTC metabolites were identified, 13 of which were confirmed based on authentic standards (Table 1). The potential metabolites were identified through comparison with the two controls (see detail in Section 2.5 and Supplementary Figure S3). Their detailed information, including retention times (RT), tentative identification, molecular formulas, theoretical mass, measured mass, and corresponding mass errors (Δ PPM), as well as the characteristic MS/MS fragment ions, are listed in Table 1.

Based on their chemical structures, the transferred metabolites were classified into two categories, namely flavonoid metabolites and phenolic catabolites. Compounds in the class of flavonoid metabolites had the characteristic carbon skeleton C6-C3-C6. A total of 14 flavonoid metabolites were characterized after fecal fermentation. The peak areas of M1, M2, and M3 in the 2 h fermentation sample experienced a significant increase compared to those in the 0 h unfermented sample, suggesting that the fecal microbiota formed them from EGCG. Based on the corresponding authentic standards, M1, M2, M3, M4, and M5 were identified as EGC, ECG, EC, gallic acid (GA), and pyrogallol (PG), respectively. For the other metabolites, due to the absence of corresponding commercial standards, their identification was carried out through comparison with the data obtained from the mentioned reference compounds and the published literature.

The MS/MS fragmentation of the detected flavonoid metabolites experienced typical retro-Diels–Alder (RDA) reactions, in line with the findings of the reported studies [14,21]. Taking deprotonated EC as an example (Figure S4), we fragmented metabolites into product ions at *m*/*z* 125, 151, and 137 using the RDA reaction, which could be considered as diagnostic product ions for EC and its derivates. In particular, the MS/MS fragmentation of EGCG, ECG, and ECG and their corresponding conjugates also gave the ions signal at *m*/*z* 125 and 137, yielded via an RDA reaction similar to GTCs.

The metabolite M6, with an elution time of 8.64 min, exhibited a precursor ion at *m*/*z* 455.0621, 2 Da (H_2_) less than that of EGCG. The molecular composition of M6 was determined to be C_22_H_16_O_11_, indicating that M6 might be a dehydrogenation metabolite of EGCG. The major fragment ions at *m*/*z* 125.02444 (^1,4^A-) and 169.01413 suggested that the A-ring structure remained unchanged and the galloyl group was present. In addition, the two characteristic ions at *m*/*z* 137.02463 (^1,3^A-) and *m*/*z* 165.01904 (^1,3^B-) were detected after the RDA reaction, suggesting that dehydrogenation occurred on the B-ring. Therefore, M6 was tentatively identified as the EGCG quinone (Figure S5). Similarly, the metabolite M7 was eluted at 9.75 min and had a deprotonated molecular ion at *m*/*z* 303.05112, providing an element composition of C_15_H_12_O_7_, 2 Da (H_2_) less than that of EGC. Several dominant ions noted at *m*/*z* 125.02444, 137.02463, and 149.01947 were observed via RDA reactions. These results agreed with those of the cleavage pathways of M6. Therefore, M7 was tentatively identified as the EGC quinone (Figure S6).

The metabolite M8 (C_22_H_16_O_10_) was eluted at 8.64 min and had a deprotonated molecular ion at *m*/*z* 439.067021, 2 Da (H_2_) less than that of ECG. The two abundant fragment ions were identified at *m*/*z* 125.02437 (^1,4^A-) and 169.01920, suggesting the presence of M8 on the A-ring structure and in the galloyl group. In addition, two strong ions at *m*/*z* 137.02455 (^1,3^A-) and *m*/*z* 149.02425 (^1,3^B-) appeared in the secondary mass spectrum of M8 after the RDA reaction, implying that the B ring was oxidized. Therefore, M7 was tentatively identified as the ECG quinone (Figure S7). The metabolite M9 was detected at *m*/*z* 287.05615 and eluted at 8.22 min, and we determined it to be C_15_H_12_O_8_. Some representative ions at *m*/*z* 125.02450, 137.02452, 149.02425, and 269.04572 appeared in the secondary mass spectrum after the loss of H_2_O and the RDA reaction, implying that the M9 was an oxidation metabolite, with the B ring of EC being oxidized. Thus, M9 was recognized, and it is shown in Figure S8.

The metabolite M10 (C_22_H_18_O_12_) was eluted at 8.67 min with deprotonated molecular ions at *m*/*z* 473.07169, 16 Da larger than that of EGCG, indicating that M10 could be an oxygenated metabolite of EGCG. In the MS/MS spectrum, the prominent ions at *m*/*z* 125.02402 and 169.01385 were produced after the RDA reaction and galloyl group cleavage, respectively. Moreover, the characteristic ion at *m*/*z* 165.01817 was detected via C-ring 1,4-cleavage, suggesting that the oxidation reaction occurred on the B-ring. Therefore, M10 was tentatively identified as the mono-oxygenated EGCG (EGCG + O).

The metabolites M11 and M12 (C_15_H_16_O_6_) were eluted at 8.40 and 8.80 min, respectively, and both were detected at *m*/*z* 291.08752 and 291.08746, 2 Da larger than that of EC, suggesting that C-ring cleavage metabolites were produced. The unique ions at *m*/*z* 247.09755 and 205.08794 in the secondary mass spectrum were detected based on the loss of CO_2_ and CO_2_ + CH_2_O_2_, respectively. In addition, the typical fragment ions at *m*/*z* 167.03503 and 139.08370 were produced via the cleavage of the A-ring, and the ion at *m*/*z* 123.04530 was produced after dropping C_2_H_4_O based on the ion at *m*/*z* 167.03503. The Clog *p* values of M11 and M12 calculated using ChemDraw 14.0 were −0.305 and −0.375, respectively. Hence, the M11 and M12 metabolites were identified as 1-(3′,4′-dihydroxyphenyl)-3-(2″,4″-trihydroxyphenyl)-2-propanol and 1-(3′,5′-dihydroxyphenyl)-3-(2″,4″-trihydroxyphenyl)-2-propanol, respectively.

The metabolite M13, detected at *m*/*z* 275.09244 and eluted at 9.97 min, was deduced as C_15_H_16_O_5_, 16 Da smaller than M11 and M12, suggesting that M13 might be a dehydroxylation metabolite of M11 or M12. In the secondary mass spectrum, a series of prominent ions at *m*/*z* 231.10236 and 189.09196 were obtained after the loss of CO_2_ and CH_2_O_2_, implying that one hydroxyl group was dropped from the C ring. Therefore, the M13 metabolite was tentatively identified as 1-(3′-hydroxyphenyl)-3-(2″,4″,6″-trihydroxy phenyl)-2-propanol or 1-(4′-hydroxyphenyl)-3-(2″,4″,6″-trihydroxyphenyl)-2-propanol.

The M14 metabolite, detected at *m*/*z* 307.08228 and eluted at 7.82 min, was deduced as C_15_H_16_O_7_, and its molecular weight (MW) was larger, by 16 Da, compared with M11 and M12, suggesting that oxidized metabolite was produced. In the secondary mass spectrum, several significant ions were observed at *m*/*z* 139.04012, 167.03503, 125.02448, 263.22664, and 221.08154. The predominated ions at *m*/z 263.22664 and 221.08154 in the secondary mass spectrum were detected based on the losses of CO_2_ and CH_2_O, respectively, implying that one hydroxyl group was added to the C ring. Therefore, the M14 metabolite was tentatively identified as 1-(3′,4,5′-dihydroxyphenyl)-3-(2″,4″,6″-trihydroxyphenyl)-2-propanol.

The metabolite M15, with an elementary formula of C_22_H_20_O_10_, was detected at 7.22 min in the XIC and *m*/*z* 443.09628 in the MS spectrum; this is 2 Da larger than that of ECG, indicating that M15 might experience a C-ring cleavage reaction. It generated a few diagnostic ions at *m*/*z* 169.01437 and 291.07657 after the fragmentation of the galloyl group. Furthermore, some representative ions at *m*/*z* 125.02464, 245.08110, and 151.04018 appeared in the secondary mass spectrum, which are the same as the cleavage pathways of M11 and M12. Thus, M15 was tentatively identified as 1-(3′,4′-dihydroxyphenyl)-3-(2″,4″′-trihydroxyphenyl)-2-propyl gallate.

The metabolite M16 (C_22_H_20_O_7_) was eluted at 14.01 min, presenting a deprotonated molecular ion of *m*/*z* 395.11353, 48 Da (O_3_) smaller than M15, indicating that M14 might be a reductive metabolite of M15. The unique ions at *m*/*z* 205.08764 in the secondary mass spectrum were detected based on the loss of CO_2_ + CH_2_O_2_. In addition, the typical fragment ions at *m*/*z* 167.03506 and 139.02444 were produced via the cleavage of the A-ring. The characteristic ion *m*/*z* 121.02946 is presumably formed via the removal of three hydroxyl groups from the galloyl group. Based on the above information, the possible chemical structure of M16 is shown in Figure S9.

The metabolites M17, M18, and M19, with a molecular formula of C_11_H_12_O_3_, were eluted at 9.15, 9.83, and 10.81 min, respectively, and they were detected at *m*/*z* 191.07140, 191.07147, and 191.07126. For the MS2 spectra, they exhibited similar fragment ions, and the primary fragment ion *m*/*z* 147.08154 formed after the loss of CO_2_. Additionally, the predominant ions at *m*/*z* 106.04253, 121.02972, 102.02972, and 107.04990 were detected, consistent with the results reported in the previous literature [21]. Furthermore, the Clog *p* value of M17 was 0.567. Unfortunately, the Clog *p* values of M18 and M19 were both 0.6226, meaning that the chemical structures of M18 and M19 could not be distinguished. Thus, M17, M18, and M19 were tentatively identified as 5-(2′-trihydroxyphenyl)-γ-valerolactone or its isomers, which could not be identified due to insufficient information being obtained.

The isomers of M20 and M21 were eluted at 5.80 and 9.19 min and detected at *m*/*z* 207.06625 and 207.06621, respectively, in the MS spectra. The molecular formulas were determined to be C_11_H_12_O_4_, 16Da (O) larger than that of M17. The MS2 spectra of M20 and M21 presented staple ions at *m*/*z* 163 and 123 via the continuous dropping of CO_2_ and C_4_H_5_O_2_. Moreover, the Clog *p* values of M20 and M21 were calculated as −0.0500000 and −0.019999, respectively. Therefore, M20 and M21 were determined to be 5-(3′,5′-dihydroxyphenyl)-γ-valerolactone and 5-(3′,4′-dihydroxyphenyl)-γ-valerolactone, respectively.

The metabolite M22 (C_11_H_14_O_5_) was eluted at 7.85 min with a deprotonated molecular ion of *m*/*z* 223.06125. Its MW was 16 Da (O) higher than those of M20 and M21, suggesting that M22 may be the mono-oxidized metabolite of M20 or M21. The predominated ions at *m*/*z* 179.07138 in the secondary mass spectrum were detected based on the loss of CO_2_. In addition, the ion at *m*/*z* 243.06890 was produced after dropping H_2_O based on the ion at *m*/*z* 271.0617. Moreover, the other fragment ions at *m*/*z* 122.03753 and 123.04522 were all identical to M20 and M21. Based on the above information, M22 was tentatively identified as 5-(3′,4′,5′-trihydroxyphenyl)-γ-valerolactone.

The metabolite M23 (C_11_H_16_O_3_) was eluted at 12.01 min with a deprotonated molecular ion of *m*/*z* 193.08691, and its MW was 2 Da (H_2_) larger than that of M17, suggesting that reduction metabolites were produced. Some representative ions at *m*/*z* 175.07658 and 149.09723 appeared in the secondary mass spectrum after the loss of H_2_O and CO_2_, implying that M23 was a phenolic metabolite. In addition, the ion at *m*/*z* 121.02948 was produced after the dropping of CO based on the ion at *m*/*z* 149.09723. Therefore, M23 was identified as 5-(2-hydroxyphenyl)-γ-valeric acid or its isomers.

Two chromatographic peaks of M24 and M25 in the XIC were eluted at 9.15 and 9.81 min, respectively, and they had an identical theoretical molecular formula: C_11_H_16_O_4_. The deprotonated molecular ions were found at *m*/*z* 209.08182 and 209.08203, 16 Da (O) larger than the size of M23, suggesting that M24 and M25 were mono-oxidized metabolites. The prominent fragment ions at *m*/*z* 191.07146, 165.09203, and 147.08165 were generated after the sequential loss of H_2_O, CO_2_, and CO. The Clog *p* values of M24 and M25 were 1.157 and 1.477, respectively, and they were calculated using ChemDraw 14.0. Hence, M24 and M25 were determined to be 5-(3′,4′-dihydroxyphenyl)-γ-valeric acid and 5-(3′,5′-dihydroxyphenyl)-γ-valeric acid, respectively.

The metabolite M26 (C_11_H_16_O_5_) was eluted at 7.85 min with a deprotonated molecular ion of *m*/*z* 225.06125, 32 Da larger than M23, indicating that M26 may be a dioxidized metabolite of M23. At the same time, other mass information was found at *m*/*z* 179.07138 and 123.04522 through measuring the loss of CO_2_ and the RDA reaction. Thus, M26 was identified as 5-(3′,4′,5′-trihydroxyphenyl)-γ-valerolactone.

Small phenolic acid compounds, products of catechin skeleton cleavage, comprise downstream metabolites. As shown in Table 1, we detected nine phenolic acid metabolites in the fermented catechin samples. A standard substance comparison allowed the identification of M28, M29, M30, M31, M33, M34, and M35 as 4-phenylbutyric acid, 3-phenylpropionic acid, 3-(3′,4′-dihydroxyphenyl) propionic acid, phenylacetic acid, 3-hydroxyphenylacetic acid, and 4-hydroxybenzoic acid, respectively. Based on their MS/MS fragmentation patterns and previously reported results, we tentatively identified M27 and M32 as 4-hydroxyphenylbutyric acid and 4-hydroxyphenylacetic acid, respectively. Phenolic acid metabolites possess a shared carboxyl group and exhibit characteristic ions in their fragmentation processes, stemming from the neutral loss of CO_2_ (44 Da).

### 3.2. Microbial Biotransformation Pathways of GTCs during In Vitro Human Fecal Fermentation

**Figure 1:**
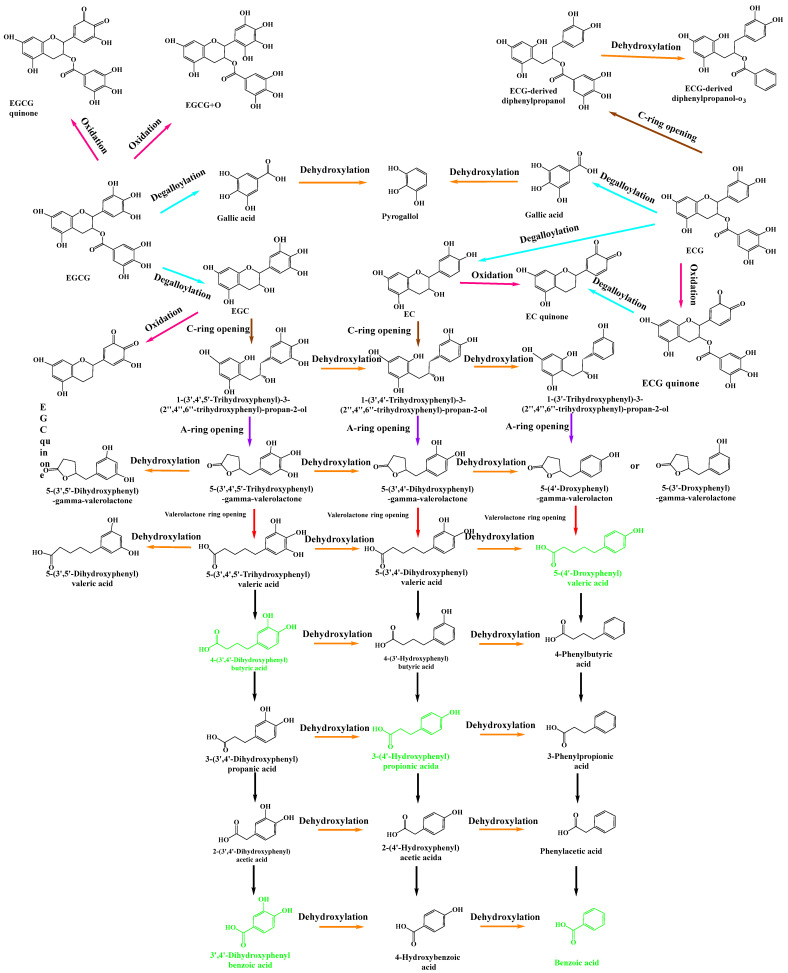
Microbial degradation pathways via which the human gut microbiota fermented GTCs. The compounds in black are detected metabolites, which are listed in Table 1, and the compounds in green are theoretical intermediates that were not detected.

In this study, GTCs were extensively catabolized by the human gut microbiota. Consequently, a total of 35 metabolites were identified via UHPLC-Orbitrap-MS/MS data analysis. A heat map was generated to show the dynamic changes of the peak areas of 35 GTCs metabolites (Figure S10). Table S2 shows the maximal concentrations of metabolites detected in fermentation broth with the time at which they were measured. As depicted in Figure S10, a rapid degradation was observed for EGCG (M0). After 12 h of fermentation, EGCG (M0) was almost entirely degraded. Conversely, the peak areas of EGC (M1), ECG (M2), and EC (M3) showed a trend of rising first and then decreased during the fermentation process, particularly that of ECG (M2), which reached its maximum level at 2 h and subsequently declined. The results suggested that EGCG may be metabolized by the gut microbiota into EGC (M1), ECG (M2), and EC (M3). The heat map illustrated that the metabolites of the GTCs derivatives were detected first at 2 h, and then were found to increase from 2 h to 8 h (M4, M16, etc.). In addition, some phenolic catabolites were initially detected at 12 h, implying that GTCs may degrade via C-ring cleavage and oxidation by the gut microbiota, ultimately resulting in smaller phenolic metabolites. The proposed metabolic pathways, via which the human intestinal flora ferment GTCs, are shown in Figure 1. In summary, the initial steps of biotransformation involved hydrolysis, oxidation, C-ring opening, and A-ring cleavage, forming upstream metabolites as a result, including catechin quinones, diphenylpropanols, phenylvalerolactones, phenylvaleric acids, gallic acids, and pyrogallols. Further degradation reactions include the shortening of the aliphatic chains of phenylvaleric acids and the dehydroxylation of phenyl moiety, producing downstream metabolites comprising several phenolic metabolites.

### 3.3. Dynamic Enhancements in Antioxidant, α-Glucosidase and α-Amylase Inhibitory Activity of GTCs during Fermentation

**Figure 2:**
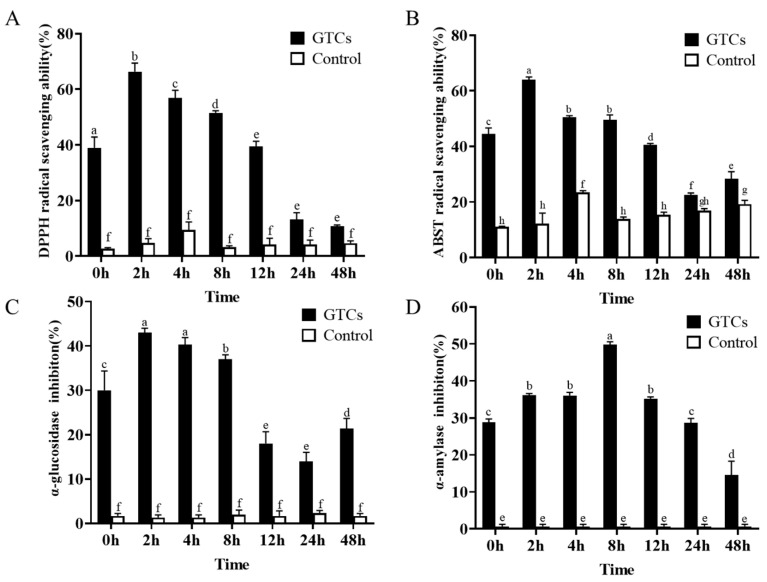
The enhancement of DPPH radical scavenging (**A**) and ABST radical scavenging abilities (**B**), α-glucosidase inhibitory ability (**C**) and α-amylase inhibitory ability (**D**) of GTCs after fermentation by the human gut microbiota. Data are expressed as mean values, and their SDs are represented by vertical bars. The presence of different letters above the group columns indicates significant differences (*n* = 3, *p* < 0.05).

The dynamic changes in the antioxidant activities of GTCs during biotransformation were investigated by evaluating the DPPH and ABTS radical scavenging abilities of the GTC samples at seven time points during fermentation. As shown in Figure 2A,B, the antioxidant capacities of the GTCs exhibited dynamic changes during fermentation. Compared to the initial time point (0 h), the DPPH radical scavenging activities of GTCs significantly increased at 2, 4, and 8 h of fermentation (*p* < 0.05), peaking at 2 h and then gradually declining (Figure 2A). Most notably, the GTCs samples also showed the highest ABTS radical scavenging activities at 2 h of fermentation (*p* < 0.05) (Figure 2B). However, due to the different mechanisms and reaction times for the assay methods, the measured DPPH and ABST radical scavenging capability values differed slightly. Regardless, fermentation by the gut microbiota can improve the antioxidant capacities of GTCs.

Inhibiting α-glucosidase and α-amylase delays the digestion and absorption of carbohydrates and glucose in the small intestine, reducing postprandial blood glucose levels and mitigating postprandial hyperglycemia [26]. Therefore, this study assessed fermentation’s impact on the inhibitory activities of GTCs against α-glucosidase and α-amylase. The results, as shown in Figure 2C,D, indicated that the α-glucosidase inhibitory capacities of GTCs were significantly enhanced at 2, 4, and 8 h compared to the initial time point (0 h) (*p* < 0.05), with the maximum inhibitory capacity achieved at 2 h. This suggests that certain bioactive compounds affecting the digested output (DO) at this stage of fermentation inhibit α-glucosidase activity. As for α-amylase inhibitory activity, the fermented samples have a greater inhibitory capacity compared to the unfermented samples, with the maximum inhibitory capacity achieved at the 8 h time point.

### 3.4. Dynamics of Microbiota during the Fermentation Process

**Figure 3:**
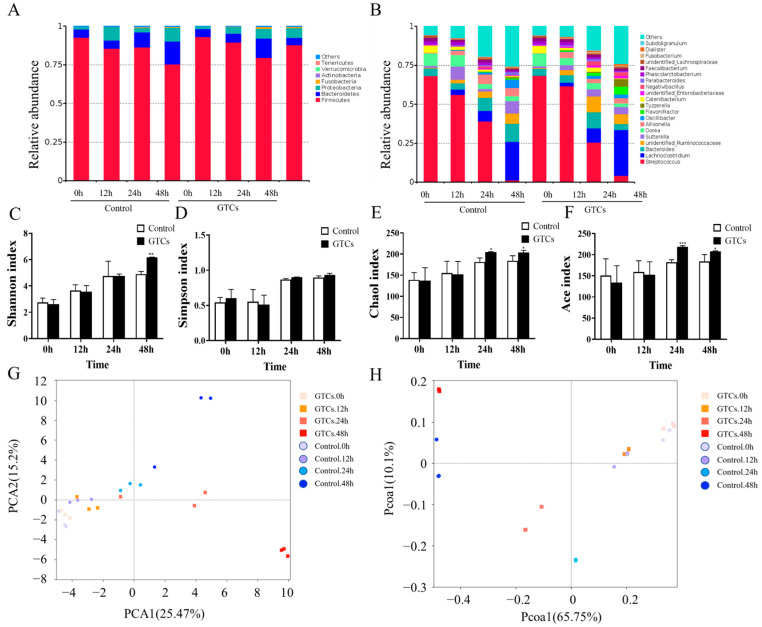
GTCs dynamically regulate the diversity and composition of human gut microbiota at each time point. (**A**) The relative abundance of gut microbiota at the phylum level. (**B**) The relative abundance of gut microbiota at the genus level. (**C**–**F**) The assessment of the alpha diversity of the gut microbiota based on different indices. (**G**,**H**) The principal coordinate analysis of the gut microbiota based on PCA and PCoA. All values are compared to the control at * *p* < 0.05, ** *p* < 0.01, and *** *p* < 0.001.

The gut microbiota composition in the fermented samples were analyzed by using 16S rRNA sequencing after 0, 12, 24, and 48 h of fermentation to evaluate the regulatory effects of GTCs on the gut microbiota. The results show that six phyla, namely *Bacteroidetes*, *Firmicutes*, *Proteobacteria*, *Fusobacteria*, *Actinobacteria*, and *Verrucomicrobiaare*, were present in the fermentation samples, and *Firmicutes* and Bacteroidetes were the dominant phyla (Figure 3A). At the genus level, 216 bacterial genera were identified across all samples. The changes in the average relative abundance of the 20 most abundant genera during fermentation are shown in Figure 3B. Interestingly, in the GTCs samples and the control, a decrease in *Bacteroides* and an increase in *Lachnoclostridium* were observed.

Furthermore, GTC incubation significantly increased the relative abundances of *Unidentified_Ruminococcaceae*, *Lachnoclostridium*, and *Flavonifractor* OTUs compared to the control after 48 h of fermentation (*p* < 0.05). Thus, the supplementation of GTC for 48 h significantly affected the microbiota’s composition. When investigating the effects of GTCs on the α-diversity of human gut microbiota, including the Shannon index, the Simpson index, and the Chao 1 and Ace indexes, we found that, after 48 h of fermentation, the Shannon, Chao 1, and Ace indices in the GTCs group significantly increased compared with the control group (Figure 3A). Based on principal component analysis (PCA) and principal coordinate analysis (PCoA), we identified a clear difference between the GTC group and the control group after 24 h of fermentation. After 48 h, the difference between the two groups became more pronounced, with the gut microbiota compositions and structures significantly differing. These results indicate that GTC supplementation increased the gut microbiota diversity and modulated the composition of the gut microbiota.

**Figure 4:**
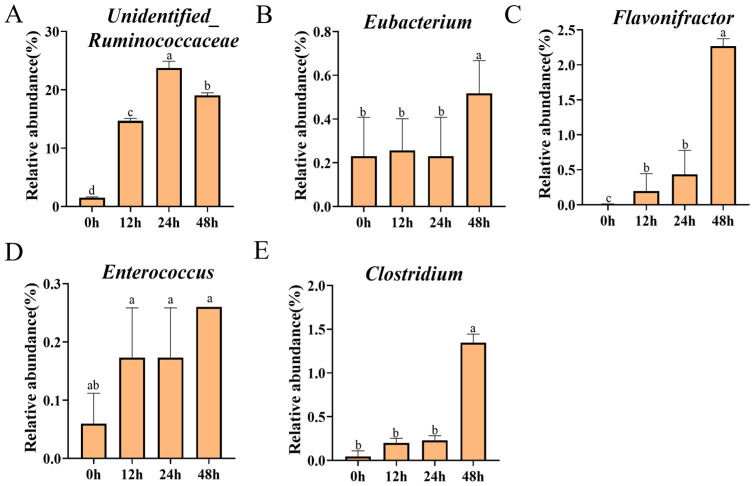
The enhancement of certain genera of gut microbiota with the ability to catabolize flavan-3-ols during fermentation at time points of 0, 12, 24, and 48 h. *Unidentified_Ruminococcus* (**A**), *Eubacterium* (**B**), *Flavonifractor* (**C**), *Enterococcus* (**D**), and *Clostridium* (**E**), (**A**–**E**) are represented by vertical bars. The presence of different letters above group columns indicates significant differences (*p* < 0.05).

To understand the interactions between the gut microbiota and biotransformation of polyphenols, we further explored the dynamic changes in specific microbials during the fermentation process. As shown in Figure 4, microbiota members able to catabolize flavan-3-ols, including *Unidentified_Ruminococcus*, *Eubacterium*, *Enterococcus*, *Clostridium*, and *Flavonifractor*, showed a dramatic increase due to the fermentation of GTCs, particularly for the 48-h-fermentated GTC sample, compared to the GTC sample at 0 h (*p* < 0.05). This result indicates that the GTCs had enrichment effects on certain genera of gut microbiota, which could simultaneously be catabolized GTCs.

## 4. Discussion

Green tea catechins (GTCs) are the main bioactive compounds of green tea. In recent years, the potential health benefits of green tea catechins and their bioactive metabolites have attracted considerable attention [26]. Previous studies have shown that GTCs have a wide range of biological activities and health effects, including anti-oxidative, anti-inflammatory, anti-viral, anti-cancer, anti-obesity, and neuroprotective effects [3,27,28,29]. However, GTCs are poorly absorbed in the small intestine, indicating that metabolism by the gut microbiota might play a crucial role in determining its bioactivities [29]. However, knowledge of the metabolic fates of GTCs influenced by gut microbiota in the human gut is very limited.

The human-gut-microbiota-mediated metabolism of GTCs has been investigated in previous studies. Wang et al. found that a human intestinal bacterium, *Eubacterium* (E.) sp. strain SDG-2, had the ability of p-dehydroxylation in the B-ring of (3R)-flavan-3-ols, such as (−)-catechin, (−)-EC, (−)-gallocatechin, and (−)-EGC, but not of (3S)-flavan-3-ols, such as (+)-catechin and (+)-EC [30]. Meselhy et al. investigated the biotransformation of (−)-ECG and related compounds using a human fecal suspension. A total of fifteen metabolites were isolated. Of these, four compounds were new. (−)-EC, (−)-EGC and their 3-O-gallates were extensively metabolized by a human fecal suspension after incubation for 24 h [31]. Using the HPLC method, Takagaki et al. determined the degradation profile of EGCG when co-incubated with rat intestinal flora, finding that EGCG was extensively metabolized into EGC and GA [32]. With the extension of fermentation time, EGC could be further converted into 1-(3′,4′,5′-trihydroxyphenyl)-3-(2″,4″,6″-trihydroxyphenyl) propan-2-ol, 1-(3′,5′-dihydroxyphenyl)-3-(2″,4″,6″-trihydroxyphenyl) propan-2-ol, and 5-(3,5-dihydroxyphenyl)-4-hydroxyvaleric acid by fecal microorganisms. However, the corresponding downstream metabolites were not further identified [32]. Stoupi et al. investigated the human fecal microbial metabolism of EC using LC-MS technology [33]. EC was found to be metabolized into 1-(3′,4′-dihydroxyphenyl)-3-(2″,4″,6″-trihydroxyphenyl)propan-2-ol and 1-(4′-dihydroxyphenyl)-3-(2″,4″,6″-trihydroxyphenyl)propan-2-ol, both of which further decomposed at the A-ring and produced phenyl-γ-valerolactones (PVLs) and phenylvaleric acids (PVAs). The data also confirmed that catabolism favored the removal of the 4′-hydroxyl rather than the 3′-hydroxyl group and that both beta-oxidation and alpha-oxidation occurred [33], which can also explain our results. In another study, Liu et al. detected the human-intestinal-microbe-associated metabolic profile of EGCG via high-resolution LC-MS/MS. A total of 14 potential microbial metabolites were identified. EGCG was promptly degraded into a series of phenolic acid metabolites, including 4-phenylbutyric acid, 3-(3′,4′-dihydroxyphenyl)propionic acid, and 3-(4′-hydroxyphenyl) propionic acid [20]. Indeed, as well as EGCG and EC, GTCs also contain ECG and EGC. Moreover, EGCG, ECG, EGC, and EC are the most abundant polyphenols in green tea, forming 30–42% of the solid green tea extract [8]. However, knowledge of the how and what microbiota-transferred metabolites of GTCs in the human gut microbiota remains very limited. In this study, these four most abundant polyphenols in green tea were used to mimic green tea polyphenols. In addition, a new UHPLC-Q-Orbitrap-MS/MS method was established to detect and characterize the metabolites produced by the human gut microbiota when fermented with GTCs. A total of 35 potential metabolites (M1–M35) of GTCs were preliminarily identified. This number is considerably higher than those in previous studies; apart from 2-(4′-hydroxyphenyl)-acetic acid and 3-hydroxybenzoic acid, all other metabolites were absent from the control samples, which do not add GTCs. Therefore, these two metabolites were presumably derived from other food phenolic substances in human feces. However, their relative abundances were much lower (less than 10%) compared to those of the samples treated with GTCs. Additionally, five structures (M6, M7, M8, M9, and M10) were identified for the first time in GTCs fermented by the human gut microbiota. Based on these metabolites, more comprehensive microbial-mediated GTCs metabolic pathways were proposed, as shown in Figure 2. In general, the transformations can be grouped into four major processes: (1) oxidation for quinone, (2) galloyl ester hydrolysis, (3) C ring opening, and (4) the further modification of the reaction products via lactonization, decarboxylation, dehydroxylation, and oxidation reactions.

To evaluate the function of the biotransformation of GTCs by the human gut microbiota’s antioxidant activity, the inhibition degrees for α-amylase and α-glucosidase at various time points in the fermentation process were measured. In this study, we found that the biological activities involved in the biotransformation of GTCs underwent significant enhancement after fermentation by the human gut microbiota. GTCs are strong antioxidants that scavenge free radicals and prevent the formation of reactive oxygen species (ROS) by chelating metal ions [34]. The study found, by comparing the pre-fermentation GTCs samples at 0 h, that the antioxidant activity of the post-fermentation GTCs significantly increased between 2 h and 8 h, indicating that GTCs’ metabolites possess significantly greater antioxidant properties than the original GTCs. Recent studies have shown that the C-ring opening catechin product 1-(3′,4′-dihydroxyphenyl)-3-(2′,4′,6′-trihydroxyphenyl)propane-2-ol exhibits higher antioxidant activity than intact catechin [35]. Our data showed that the C-ring opening metabolites of GTCs (M11–M15) have larger peak areas during the fermentation time of 2–8 h (Table S1), indicating that M11–M15 may contribute to the enhancement of fermented GTCs. Takagaki et al. reported that the metabolites PVLs (M18–M22), cyclic cleavage metabolites of GTCs, displayed better free radical scavenging abilities than the parent catechins [32]. Furthermore, the microbial metabolites of GTCs (M4–M10) also showed larger peak areas between 2 and 8 h of fermentation, indicating potential significant antioxidant properties (Table S1). It has been reported that gallic acid (M5) and pyrogallol (M6) also displayed substantial antioxidant activities [36]. Therefore, we speculated that these metabolites increase the antioxidant activity of the GTCs, as observed during fermentation from 2 to 8 h. Our data indicated that biotransferred GTCs may serve as antioxidant reagents. α-amylase and α-glucosidase are two essential carbohydrase enzymes related to the postprandial hyperglycemic effect [37]. Previous studies have reported that GTCs had stronger inhibitory effects on these enzymes [38]. Notably, our chemical analysis data showed that the α-amylase and α-glucosidase inhibitory abilities of the GTCs sample increased after fermentation compared to the initial without-fermentation sample, indicating that the products of microbial metabolites strongly inhibit α-amylase and α-glucosidase. Several studies have reported the antihyperglycemic effects of gallic acid [39]. The results suggested that the fermented GTCs might be used as dietary supplement to prevent diabetes and obesity. However, the exact GTC metabolites participating in enzyme inhibition need to be further investigated in a future study.

A growing body of evidence suggests that the interaction of tea polyphenols (TPP) with the gut microbiota leads to metabolites playing an important role in improving human health, though this interaction is not fully understood [40,41]. α-diversity represents the biodiversity of the gut microbiota. Previous studies have demonstrated that TPP can normalize the perturbed a-diversity caused by HFD and other challenges [42]. Our data showed that incubating GTCs with the gut microbiota significantly enhanced the α-diversity of the modulatory gut microbiota compared to those of the control samples. Notably, our results suggested that the gut microbiota needed time to adapt to the presence of GTCs (up to 12 h). After 12 h of fermentation, the composition of gut microbiota was altered by GTCs supplementation. Previous studies have reported that specific *Flavonifractor*, one of the most extensively researched flavonoid-degrading gut bacteria, is a core genus present in the human gut microbiota. It has been reported that flavanone- or flavanonol-cleaving reductases from *Flavonifractor plautii* degrade-specific flavonoids, enabling the C-ring cleavage of EC [39]. Our study found that the fermentation of GTCs substantially elevated the relative abundance of *Flavonifractor*, particularly at the 48 h of fermentation. Similarly, past research demonstrated that *Unidentified_Ruminococcus*, *Eubacterium*, *Enterococcus*, and *Clostridium* can participate in the metabolism of flavonoids, subsequently generating small phenolic acids through the lactonization and dehydroxylation of flavonoids [42]. Consequently, these increased specific intestinal bacteria that may promote the further metabolism of GTCs. We realized that our in vitro fecal fermentation model imitates the colonic digestion. However, this in vitro method cannot model most of the processing of the human diet as digested in the small intestine [43].

## 5. Conclusions

In conclusion, our results showed that the main GTCs were prone to be metabolized by the human gut microbiota via in vitro fermentation. This study identified a total of 35 metabolites of GTCs fermented by the human gut microbiota, far more than those identified in previous reports. Of which, five metabolites, namely the EGCG quinone, EGC quinone, ECG quinone, EC quinone, and mono-oxygenated EGCG, were identified for the first time in fermented GTC produced by the human gut microbiota. Furthermore, all their corresponding metabolic pathways were outlined. Notably, the antioxidant, α-amylase, and α-glucosidase inhibitory activities of the GTCs samples increased after fermentation compared to those of the initial sample. An array of GTC-transferred metabolites that accumulated at 2–8 h of fermentation may contribute to this enhancement activity. Additionally, the GTCs significantly altered the diversity and composition of the gut microbiota and enriched certain gut microbiota, which may be involved in the further transformation of GTCs. These findings provide new insights for understanding the microbial metabolism and health benefits of green tea catechins.

## Figures and Tables

**Table 1:** UHPLC-Q-Orbitrap-MS/MS-based identification of the fermentated metabolites of GTCs by human fecal in vitro.

No.	RT.	Tentative Identification	Formula	Theoretical Mass *m*/*z*	Experimental Mass *m*/*z*	Error (ppm)	MS/MS Fragments
M0	8.62	EGCG ^a^	C_22_H_18_O_11_	458.08545	458.08546	0.02	125.02437, 169.01418
M1	7.64	EGC ^a^	C_15_H_14_O_7_	306.07450	306.07447	−0.10	125.02433, 137.02422, 167.03477, 121.02950, 109.02927
M2	9.74	ECG ^a^	C_22_H_18_O_10_	442.09054	442.09046	−0.18	125.02451, 169.01428, 109.0259, 231.06375
M3	8.66	EC ^a^	C_15_H_14_O_6_	290.07958	290.07963	0.17	109.02954, 123.04526, 245.08153, 151.04005
M4	3.45	gallic acid ^a^	C_7_H_6_O_5_	170.02207	170.02204	−0.18	169.01439, 125.02456, 69.03470, 97.02968, 79.01898
M5	3.67	Pyrogallol ^a^	C_6_H_6_O_3_	126.03224	126.03230	0.48	125.02448, 97.02959, 81.0364, 79.01917, 69.03465
M6	7.64	EGCG quinone	C_22_H_16_O_11_	456.06926	456.06992	1.45	125.02444, 169.01413, 137.02463, 149.01947, 285.04010, 161.02415
M7	9.75	EGC quinone	C_15_H_12_O_7_	304.05830	304.05894	2.10	137.02446, 125.02437, 165.01947, 137.02453, 285.04010, 303.0507
M8	8.61	ECG quinone	C_22_H_18_O_10_	440.07435	440.07484	1.11	125.02437, 137.02455, 161.02403, 169.01920, 149.02425
M9	8.67	EC quinone	C_15_H_12_O_6_	288.06338	288.06397	2.05	125.02450, 161.02403, 137.02452, 149.02425, 269.04572
M10	14.01	EGCG+O	C_22_H_18_O_12_	474.07983	474.07951	−0.67	125.02402, 165.01817, 169.01385
M11	8.73	1-(3′,4′-Dihydroxyphenyl)-3-(2″,4″,6″-Trihydroxyphenyl)-propan-2-ol	C_15_H_16_O_6_	292.09523	292.09528	0.17	123.04530, 135.04524, 167.03503, 247.09755
M12	8.40	1-(3′,5′-Dihydroxyphenyl)-3-(2″,4″,6″-Trihydroxyphenyl)-propan-2-ol	C_15_H_16_O_6_	292.09523	292.09534	0.38	123.04530, 167.03503, 247.09755, 205.08794, 139.0837, 109.02961
M13	9.97	1-(3′-droxyphenyl)-3-(2″,4″,6″-Trihydroxyphenyl)-propan-2-ol	C_15_H_16_O_5_	276.09970	276.10032	−0.22	107.05042, 231.10236, 167.07207, 189.09196, 147.08142
M14	7.82	1-(3′,4′,5′-Trihydroxyphenyl)-3-(2″,4″,6″-Trihydroxyphenyl)-propan-2-ol	C_15_H_16_O_7_	308.09015	308.09010	−0.17	139.04012, 263.22664, 167.03503, 125.02448, 221.08154
M15	7.22	1-(3′,4′-Dihydroxyphenyl)-3- (2″,4″,6″-Trihydroxyphenyl)-propan-2-yl gallate	C_22_H_20_O_10_	444.10564	444.10410	−3.47	125.02464, 169.01437, 291.07657, 245.08110, 137.02432
M16	14.01	ECG –O_3_	C_22_H_20_O_7_	396.12090	396.12049	−1.04	125.02454, 167.03506, 121.02946, 205.04964, 139.02444, 109.02969
M17	9.15	5-(2′-Hydroxyphenyl)-γ-valerolactone	C_11_H_12_O_3_	192.07919	192.07922	0.26	147.08154, 106.04253, 121.02972, 102.02972, 107.04990
M18	9.83	5-(3′-Hydroxyphenyl)-γ-valerolactone or its isomers	C_11_H_12_O_3_	192.07919	192.07929	0.51	147.08163, 105.93622, 123.02972, 102.94908, 107.05032
M19	10.81	5-(3′-Hydroxyphenyl)-γ-valerolactones or its isomers	C_11_H_12_O_3_	192.07919	192.07908	−0.59	147.08177, 106.04253, 121.02988, 102.94888, 191.07159
M20	9.28	5-(3′,4′-Dihydroxyphenyl)-γ-valerolactone	C_11_H_12_O_4_	208.07410	208.07407	−0.14	123.04532, 163.07660, 122.03742, 207.06624, 81.03461
M21	8.68	5-(3′,5′-Dihydroxyphenyl)-γ-valerolactone	C_11_H_12_O_4_	208.07410	208.07403	−0.34	123.04528, 163.07646, 122.03754, 81.03459, 79.05441
M22	7.85	5-(3′,4′,5′-Trihydroxyphenyl)-γ-valerolactone	C_11_H_12_O_5_	224.06902	224.06902	0.00	243.06890, 179.07138, 123.04522, 133.06598, 122.03753, 161.06102
M23	12.01	5-(2′-Hydroxyphenyl) -γ-valeric acid or its isomers	C_11_H_14_O_3_	194.09484	194.09475	−0.46	193.08685, 175.07658, 149.09723, 106.04269, 121.02948
M24	9.15	5-(3′,5′-Dihydroxyphenyl)-γ-valeric acid	C_11_H_14_O_4_	210.08975	210.08964	−0.52	191.07146, 165.09203, 101.02448, 107.05035, 147.08165
M25	9.81	5-(3′,4′-Dihydroxyphenyl)-γ-valeric acid	C_11_H_14_O_4_	210.08975	210.08964	−0.52	123.08171, 81.032487, 107.05025, 149.06085, 147.08154, 91.05512
M26	7.91	5-(3′,4′,5′-Trihydroxyphenyl)-γ-valeric acid	C_11_H_14_O_5_	226.08467	226.08460	−0.31	179.07138, 123.04532, 81.03457, 101.02434
M27	7.60	4-Hydroxyphenylbutyric acid	C_10_H_12_O_3_	180.07864	180.07918	2.94	179.07130, 134.98804, 90.99820, 04.92816
M28	9.28	4-phenylbutyric acid ^a^	C_10_H_12_O_2_	164.08427	164.08421	−0.37	163.07648, 121.06597, 81.03464, 145.89063
M29	11.97	3-phenylpropionic acid ^a^	C_9_H_10_O_2_	150.06862	150.06852	−0.67	149.06081, 105.07104, 123.46254, 103.05509
M30	8.81	3-(3′,4′-Dihydroxyphenyl)propanoic acid ^a^	C_9_H_10_O_4_	182.05845	182.05851	0.33	181.05078, 112.98579, 92.99387, 136.98322
M31	11.12	phenylacetic acid ^a^	C_8_H_8_O_2_	136.05297	136.05306	0.66	135.04448, 91.05541, 67.72299
M32	8.50	2-(4′-Hydroxyphenyl)acetic acid ^a^	C_8_H_8_O_3_	152.04789	152.04787	−0.13	107.05036
M33	9.84	2-(3′-Hydroxyphenyl)acetic acid	C_8_H_8_O_3_	152.04789	152.04777	−0.79	151.04015, 107.05034
M34	7.77	2-(3′,4′-Dihydroxyphenyl)acetic acid ^a^	C_8_H_8_O_4_	168.04280	168.04267	−0.77	123.04521, 95.05029
M35	7.84	4-Hydroxybenzoic acid ^a^	C_7_H_6_O_3_	138.03224	138.03216	−0.58	137.06108, 93.03466, 85.05035

^a^: Confirmation in comparison with authentic standards; RT: retention time.

## Data Availability

The original contributions presented in the study are included in the article/Supplementary Material, further inquiries can be directed to the corresponding authors.

## Supplementary Materials

The following supporting information can be downloaded at: https://www.mdpi.com/article/10.3390/foods13050792/s1, Figure S1: Chemical structures of EGCG, EGC, ECG, and EC. The compounds in red are shared same structures among EGCG, EGC, ECG, and EC; Figure S2: The workflow for the metabolic study; Figure S3: Total ion chromatograms (TIC) of control 1 using human fecal suspension (HFS) and medium (A), control 2 using GTCs incubated for 0 h by HFS (B), and GTCs samples mixed at different time points (2, 4, 8, 12, 24 and 48 h) after fermentation by HFS (C); Figure S4: The MS2 spectrum and the cleavage pathways of EC; Figure S5: The MS2 spectrum and the cleavage pathways of M6; Figure S6: The MS2 spectrum and the cleavage pathways of M7; Figure S7: The MS2 spectrum and the cleavage pathways of M8; Figure S8: The MS2 spectrum and the cleavage pathways of M9; Figure S9: The MS2 spectrum and the cleavage pathways of M16; Figure S10: Heat map of dynamic changes in the peak areas of microbial GTCs metabolites at different fermentation times of 0, 2, 4, 8, 12, 24, and 48 h. Red and blue boxes represent values that are higher and lower than the mean value, respectively. Table S1: The peak areas of metabolites detected in fermentation broth at different fermentation times at 0, 2, 4, 8, 12, 24, and 48 h, respectively; Table S2: The maximal concentrations of metabolites detected fermentation broth at different fermentation times at 0, 2, 4, 8, 12, 24, 48 h, respectively; Table S3: Basic information of volunteers.

## References

[1] Suzuki Y., Miyoshi N., Isemura M. (2012). Health-Promoting Effects of Green Tea. Proc. Jpn. Acad. Ser. B Phys. Biol. Sci..

[2] Annunziata G., Maisto M., Schisano C., Ciampaglia R., Daliu P., Narciso V., Tenore G., Novellino E. (2018). Colon Bioaccessibility and Antioxidant Activity of White, Green and Black Tea Polyphenols Extract after In Vitro Simulated Gastrointestinal Digestion. Nutrients.

[3] Butt M.S., Ahmad R.S., Sultan M.T., Qayyum M.M.N., Naz A. (2015). Green tea and anticancer perspectives: Updates from last decade. Crit. Rev. Food Sci. Nutr..

[4] Kapoor M.P., Sugita M., Fukuzawa Y., Timm D., Ozeki M., Okubo T. (2021). Green Tea Catechin Association with Ultraviolet Radiation-Induced Erythema: A Systematic Review and Meta-Analysis. Molecules.

[5] Castaldo L., Toriello M., Sessa R., Izzo L., Lombardi S., Narváez A., Ritieni A., Grosso M. (2021). Antioxidant and Anti-Inflammatory Activity of Coffee Brew Evaluated after Simulated Gastrointestinal Digestion. Nutrients.

[6] Higdon J.V., Frei B. (2003). Tea catechins and polyphenols: Health effects, metabolism, and antioxidant functions. Crit. Rev. Food Sci. Nutr..

[7] Musial C., Kuban-Jankowska A., Gorska-Ponikowska M. (2020). Beneficial Properties of Green Tea Catechins. Int. J. Mol. Sci..

[8] Kochman J., Jakubczyk K., Antoniewicz J., Mruk H., Janda K. (2020). Health Benefits and Chemical Composition of Matcha Green Tea: A Review. Molecules.

[9] Borges G., van der Hooft J.J.J., Crozier A. (2016). A comprehensive evaluation of the [2-14C](-)-epicatechin metabolome in rats. Free Radic. Biol. Med..

[10] Mena P., Bresciani L., Brindani N., Ludwig I.A., Pereira-Caro G., Angelino D., Llorach R., Calani L., Brighenti F., Clifford M.N. (2019). Phenyl-γ-valerolactones and phenylvaleric acids, the main colonic metabolites of flavan-3-ols: Synthesis, analysis, bioavailability, and bioactivity. Nat. Prod. Rep..

[11] Li Q., Van Herreweghen F., Onyango S.O., De Mey M., Van de Wiele T. (2022). In Vitro Microbial Metabolism of (+)-Catechin Reveals Fast and Slow Converters with Individual-Specific Microbial and Metabolite Markers. J. Agric. Food Chem..

[12] Al-Ishaq R.K., Liskova A., Kubatka P., Büsselberg D. (2021). Enzymatic Metabolism of Flavonoids by Gut Microbiota and Its Impact on Gastrointestinal Cancer. Cancers.

[13] Rowland I., Gibson G., Heinken A., Scott K., Swann J., Thiele I., Tuohy K. (2018). Gut microbiota functions: Metabolism of nutrients and other food components. Eur. J. Nutr..

[14] Bao T., Zhang M., Zhou Y., Chen W. (2021). Phenolic profile of jujube fruit subjected to gut microbiota fermentation and its antioxidant potential against ethyl carbamate-induced oxidative damage. J. Zhejiang Univ. Sci. B.

[15] Yan Y., Fu C., Cui X., Pei X., Li A., Qin X., Du C., Du H. (2020). Metabolic profile and underlying antioxidant improvement of Ziziphi Spinosae Folium by human intestinal bacteria. Food Chem..

[16] Kc D., Sumner R., Lippmann S. (2020). Gut microbiota and health. Postgrad. Med..

[17] Zafar H., Saier M.H. (2021). Gut Bacteroides species in health and disease. Gut Microbes.

[18] Li Y., Rahman S.U., Huang Y., Zhang Y., Ming P., Zhu L., Chu X., Li J., Feng S., Wang X. (2020). Green tea polyphenols decrease weight gain, ameliorate alteration of gut microbiota, and mitigate intestinal inflammation in canines with high-fat-diet-induced obesity. J. Nutr. Biochem..

[19] Sun L., Su Y., Hu K., Li D., Guo H., Xie Z. (2023). Microbial-Transferred Metabolites of Black Tea Theaflavins by Human Gut Microbiota and Their Impact on Antioxidant Capacity. Molecules.

[20] Liu Z., de Bruijn W.J.C., Bruins M.E., Vincken J.-P. (2020). Reciprocal Interactions between Epigallocatechin-3-gallate (EGCG) and Human Gut Microbiota In Vitro. J. Agric. Food Chem..

[21] Yin J., Ma Y., Liang C., Gao J., Wang H., Zhang L. (2019). A Systematic Study of the Metabolites of Dietary Acacetin in Vivo and in Vitro Based on UHPLC-Q-TOF-MS/MS Analysis. J. Agric. Food Chem..

[22] Wang B., Lu Y., Hu X., Feng J., Shen W., Wang R., Wang H. (2020). Systematic Strategy for Metabolites of Amentoflavone In Vivo and In Vitro Based on UHPLC-Q-TOF-MS/MS Analysis. J. Agric. Food Chem..

[23] Li M., Luo X., Ho C.-T., Li D., Guo H., Xie Z. (2022). A new strategy for grading of Lu’an guapian green tea by combination of differentiated metabolites and hypoglycaemia effect. Food Res. Int..

[24] Zahid H.F., Ali A., Ranadheera C.S., Fang Z., Ajlouni S. (2023). Identification of Phenolics Profile in Freeze-Dried Apple Peel and Their Bioactivities during In Vitro Digestion and Colonic Fermentation. Int. J. Mol. Sci..

[25] Zhang S., Mao B., Cui S., Zhang Q., Zhao J., Tang X., Chen W. (2023). Absorption, metabolism, bioactivity, and biotransformation of epigallocatechin gallate. Crit. Rev. Food Sci. Nutr..

[26] Favari C., Mena P., Curti C., Rio D., Angelino D., Tomás-Barberán F.A., González-Sarrías A., García-Villalba R. (2020). Flavan-3-ols: Catechins and Proanthocyanidins. Dietary Polyphenols.

[27] Han X.-D., Zhang Y.-Y., Wang K.-L., Huang Y.-P., Yang Z.-B., Liu Z. (2017). The involvement of Nrf2 in the protective effects of (-)-Epigallocatechin-3-gallate (EGCG) on NaAsO2-induced hepatotoxicity. Oncotarget.

[28] Absorption, Metabolism, Anti-Cancer Effect and Molecular Targets of Epigallocatechin Gallate (EGCG): An Updated Review-PubMed. https://pubmed.ncbi.nlm.nih.gov/27645804/.

[29] Cao H., Chen X., Jassbi A.R., Xiao J. (2015). Microbial biotransformation of bioactive flavonoids. Biotechnol. Adv..

[30] Wang L.Q., Meselhy M.R., Li Y., Nakamura N., Min B.S., Qin G.W., Hattori M. (2001). The heterocyclic ring fission and dehydroxylation of catechins and related compounds by Eubacterium sp. strain SDG-2, a human intestinal bacterium. Chem. Pharm. Bull..

[31] Meselhy M.R., Nakamura N., Hattori M. (1997). Biotransformation of (-)-epicatechin 3-O-gallate by human intestinal bacteria. Chem. Pharm. Bull..

[32] Takagaki A., Nanjo F. (2010). Metabolism of (-)-epigallocatechin gallate by rat intestinal flora. J. Agric. Food Chem..

[33] Stoupi S., Williamson G., Drynan J.W., Barron D., Clifford M.N. (2010). A comparison of the in vitro biotransformation of (-)-epicatechin and procyanidin B2 by human faecal microbiota. Mol. Nutr. Food Res..

[34] Zhang Q., Liu J., Duan H., Li R., Peng W., Wu C. (2021). Activation of Nrf2/HO-1 signaling: An important molecular mechanism of herbal medicine in the treatment of atherosclerosis via the protection of vascular endothelial cells from oxidative stress. J. Adv. Res..

[35] Chen W., Zhu X., Lu Q., Zhang L., Wang X., Liu R. (2020). C-ring cleavage metabolites of catechin and epicatechin enhanced antioxidant activities through intestinal microbiota. Food Res. Int..

[36] Zhang K.-Q., Lin L.-L., Xu H.-J. (2022). Research on antioxidant performance of diglucosyl gallic acid and its application in emulsion cosmetics. Int. J. Cosmet. Sci..

[37] He Q., Lv Y., Yao K. (2007). Effects of tea polyphenols on the activities of α-amylase, pepsin, trypsin and lipase. Food Chem..

[38] Zhu S., Li J., Li W., Li S., Yang X., Liu X., Sun L. (2022). Enzymic catalyzing affinity to substrate affects inhibitor-enzyme binding interactions: Inhibition behaviors of EGCG against starch digestion by individual and co-existing α-amylase and amyloglucosidase. Food Chem..

[39] Wang H., Fowler M.I., Messenger D.J., Ordaz-Ortiz J.J., Gu X., Shi S., Terry L.A., Berry M.J., Lian G., Wang S. (2021). Inhibition of the intestinal postprandial glucose transport by gallic acid and gallic acid derivatives. Food Funct..

[40] Selma M.V., Espín J.C., Tomás-Barberán F.A. (2009). Interaction between phenolics and gut microbiota: Role in human health. J. Agric. Food Chem..

[41] Yan R., Ho C.-T., Zhang X. (2020). Interaction between Tea Polyphenols and Intestinal Microbiota in Host Metabolic Diseases from the Perspective of the Gut-Brain Axis. Mol. Nutr. Food Res..

[42] Li J., Chen C., Yang H., Yang X. (2021). Tea polyphenols regulate gut microbiota dysbiosis induced by antibiotic in mice. Food Res. Int..

[43] Folz J., Culver R.N., Morales J.M., Grembi J., Triadafilopoulos G., Relman D.A., Huang K.C., Shalon D., Fiehn O. (2023). Human metabolome variation along the upper intestinal tract. Nat. Metab..

