# Supplementary material for: Microbial-Transferred Metabolites and Improvement of Biological Activities of Green Tea Catechins by Human Gut Microbiota
Source: Foods. 2024 Mar 4;13(5):792. doi: 10.3390/foods13050792 (PMC10930907; doi:10.3390/foods13050792)
Supplement: Supplementary file 1 [file foods-13-00792-s001.zip › foods-2860108-supplementary.pdf]

Figure S1. Chemical structures of EGCG, EGC, ECG, and EC. The compounds in red are shared same structures among EGCG, EGC, ECG, and EC.

Figure S2. The workflow for the metabolic study.

Figure S3. Total ion chromatograms (TIC) of control 1 using human fecal suspension (HFS) and medium (A), control 2 using GTCs incubated for 0h by HFS(B), and GTCs samples mixed at different time points (2, 4, 8, 12, 24 and 48h) after fermentation by HFS(C).

Figure S4. The MS2 spectrum and the cleavage pathways of EC.

Figure S5. The MS2 spectrum and the cleavage pathways of M6.

Figure S6. The MS2 spectrum and the cleavage pathways of M7.

Figure S7. The MS2 spectrum and the cleavage pathways of M8.

Figure S8. The MS2 spectrum and the cleavage pathways of M9.

Figure S9. The MS2 spectrum and the cleavage pathways of M16.

Figure S10. Heat map of dynamic changes in the peak areas of microbial GTCs metabolites at different fermentation times of 0, 2, 4, 8, 12, 24, and 48 h. Red and blue boxes represent values that are higher and lower than the mean value, respectively.

Table S1. The peak areas of metabolites detected in fermentation broth at different fermentation times at 0, 2, 4, 8, 12, 24, and 48 h, respectively.

Table S2 The maximal concentrations of metabolites detected fermentation broth at different fermentation times at 0, 2, 4, 8, 12, 24, 48 h, respectively.

Table S3. Basic information of volunteers.

Figure S1.

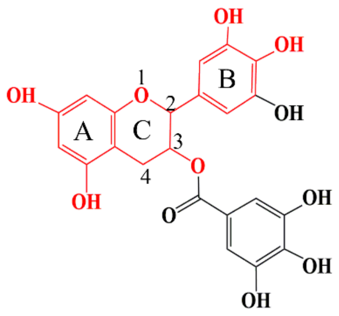

Epigallocatechin gallate (EGCG)

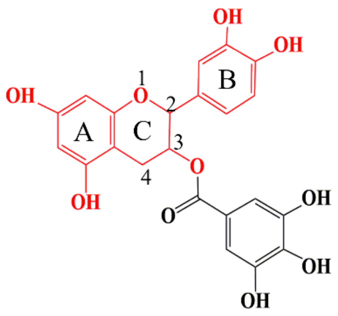

Epicatechin gallate (ECG)

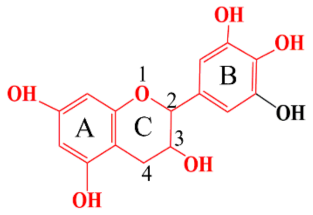

Epigallocatechin (EGC)

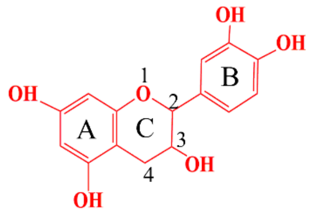

Epicatechin (EC)

Figure S2.

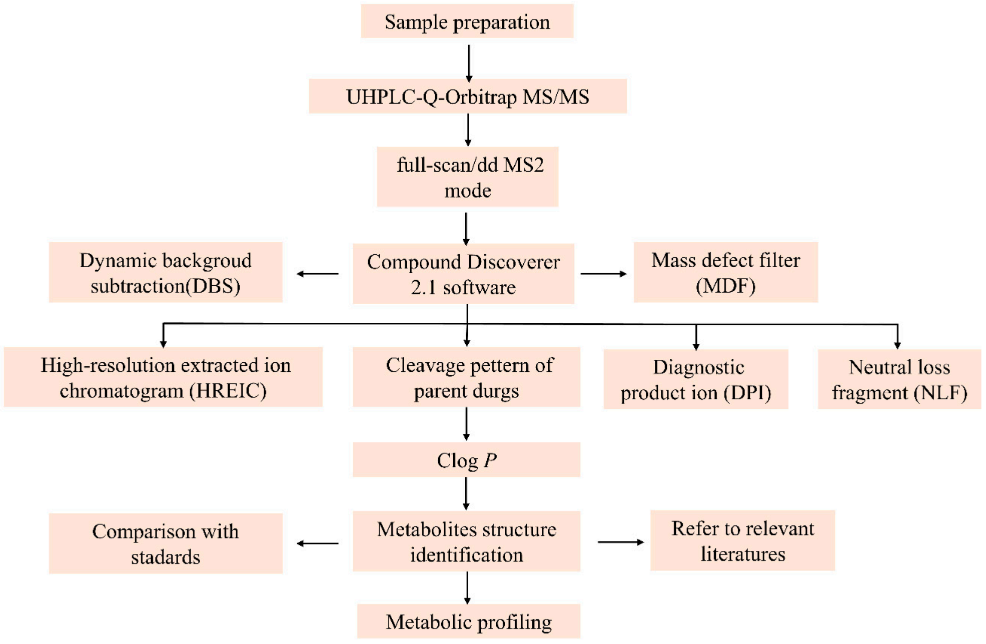

Figure S3.

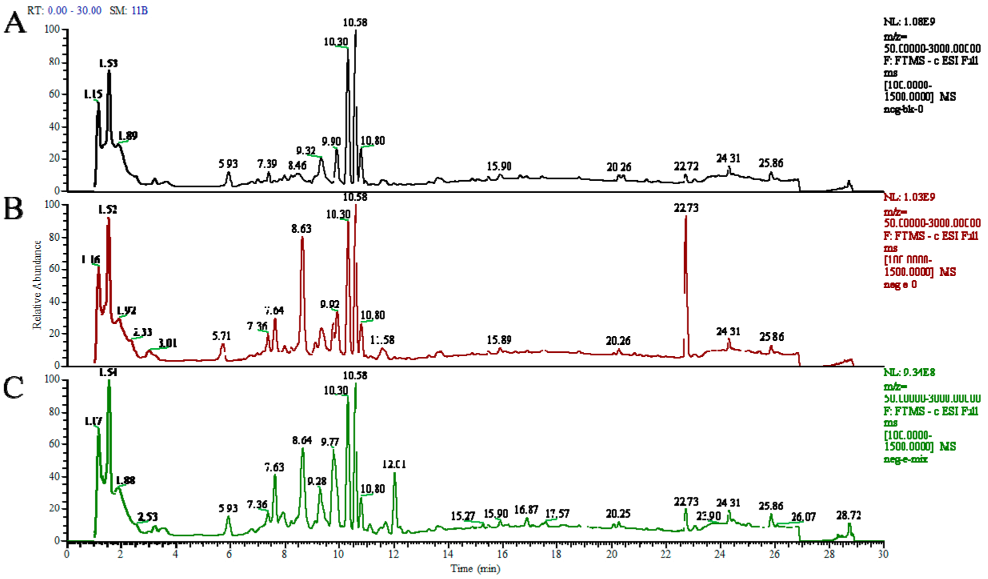
A
RT: 0.00 - 30.00 SM: 11B
NL: 1.08E9
m/z= 50.00000-3000.00000 F: FTMS - c ESI Full ms [100.0000-1500.0000] MS neg-bk-0
B
NL: 1.03E9
m/z= 50.00000-3000.00000 F: FTMS - c ESI Full ms [100.0000-1500.0000] MS neg e 0
C
NL: 9.34E8
m/z= 50.00000-3000.00000 F: FTMS - c ESI Full ms [100.0000-1500.0000] MS neg-e-mix
Relative Abundance
Time (min)

Figure S4.

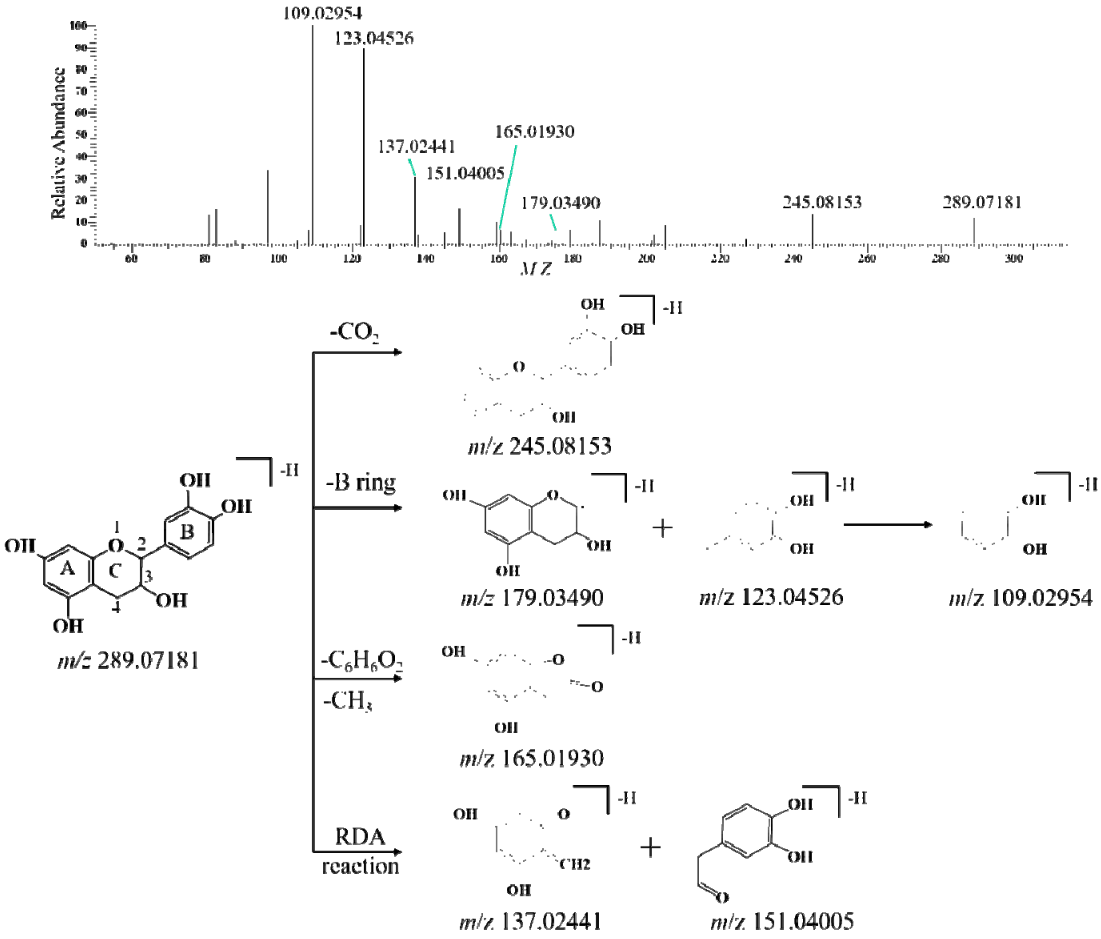
109.02954
123.04526
137.02441
165.01930
151.04005
179.03490
245.08153
289.07181
Relative Abundance
M/Z
-CO2
m/z 245.08153
-B ring
m/z 179.03490
m/z 123.04526
m/z 109.02954
A
B
C
m/z 289.07181
-C6H6O2
-CH3
m/z 165.01930
RDA reaction
m/z 137.02441
m/z 151.04005

Figure S5.

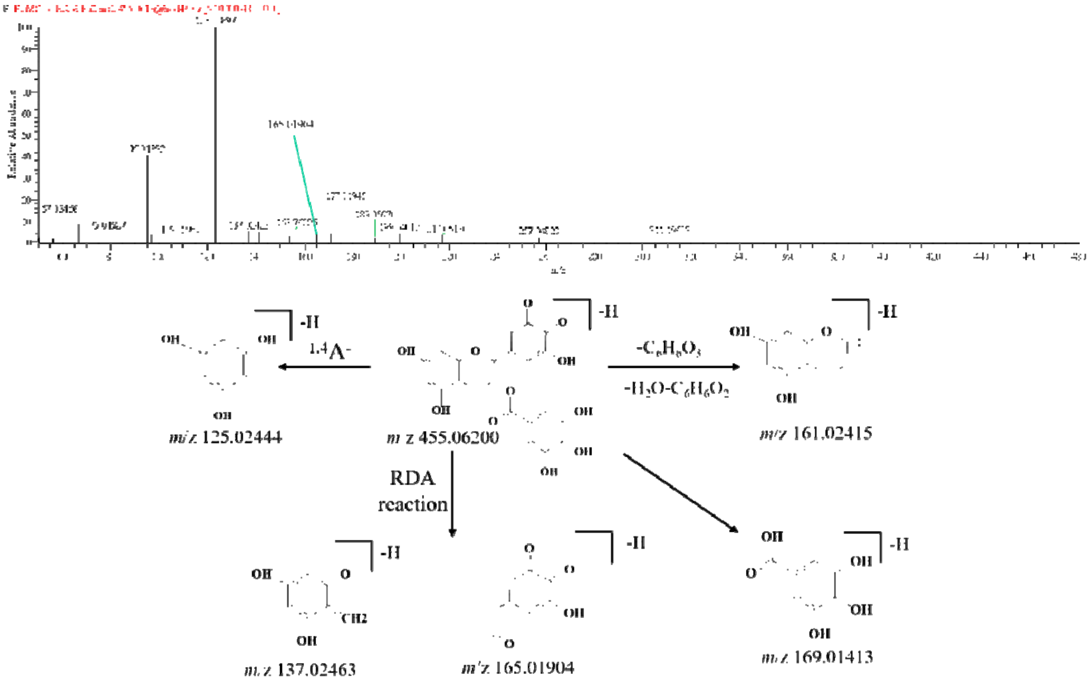

Figure S6.

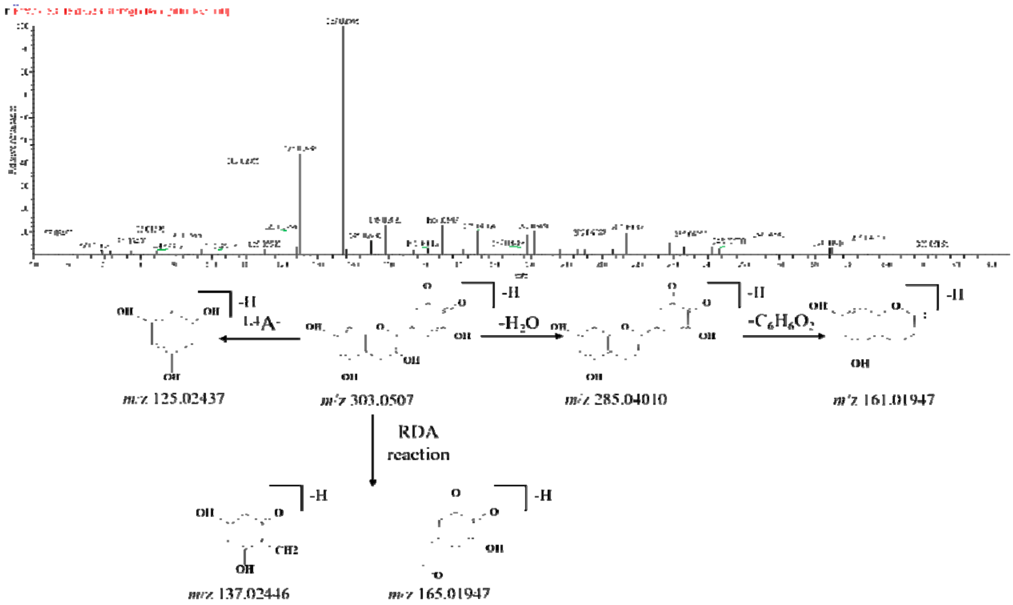

Figure S7.

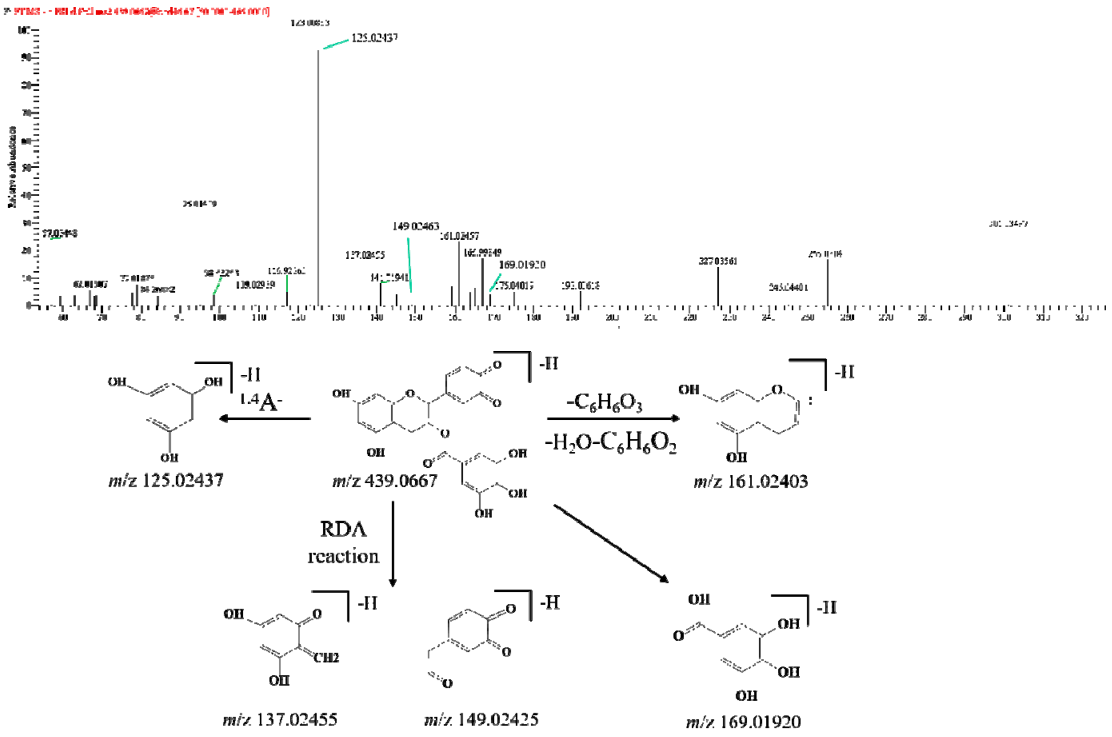

Figure S8.

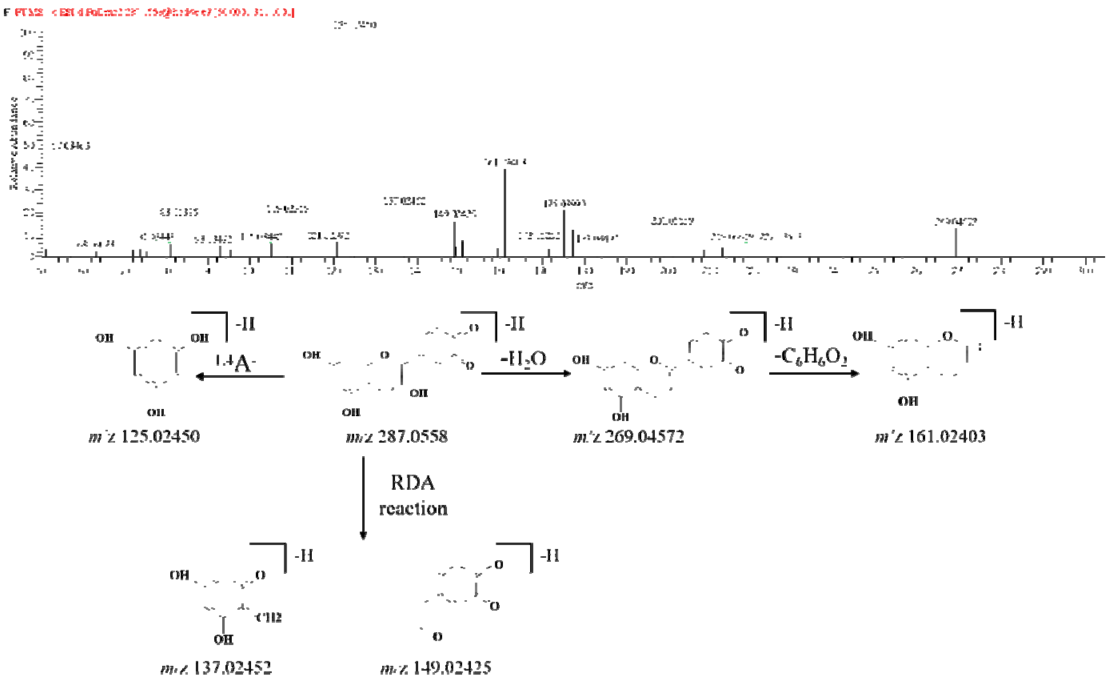

Figure S9.

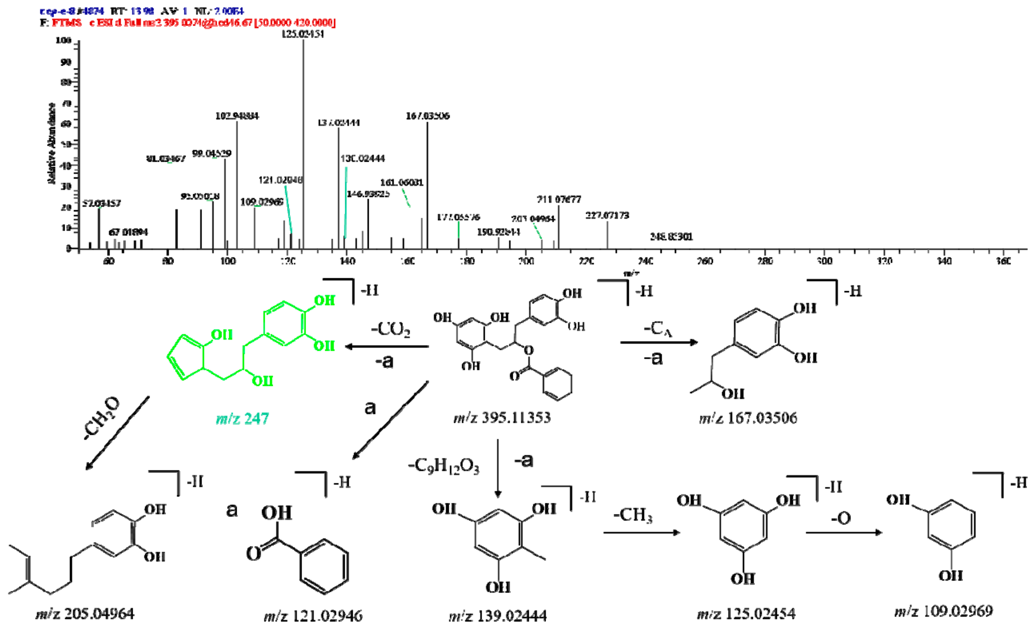

Figure S10.

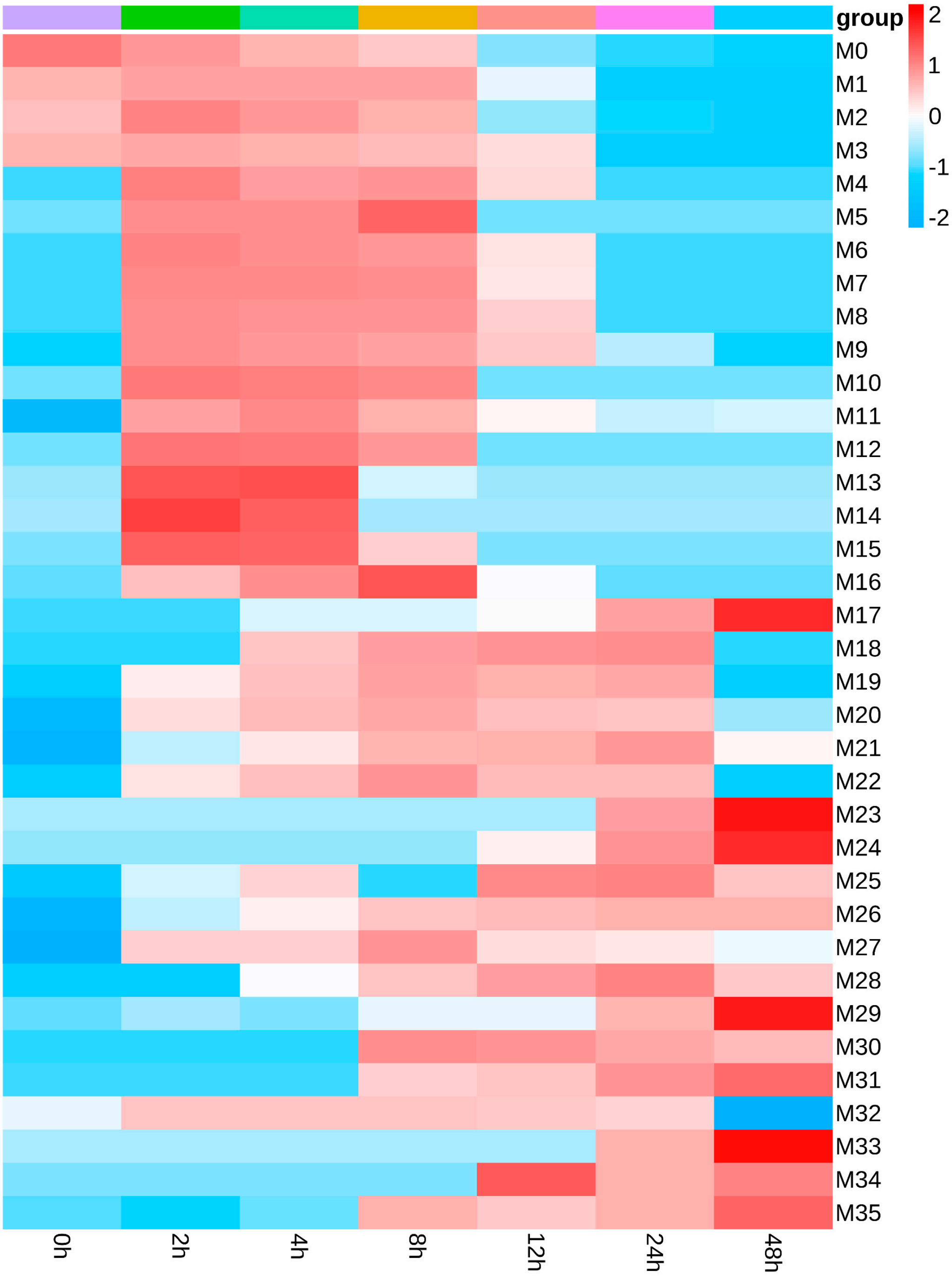
group
2
1
0
-1
-2
M0
M1
M2
M3
M4
M5
M6
M7
M8
M9
M10
M11
M12
M13
M14
M15
M16
M17
M18
M19
M20
M21
M22
M23
M24
M25
M26
M27
M28
M29
M30
M31
M32
M33
M34
M35
0h
2h
4h
8h
12h
24h
48h

Table S1 The peak areas of metabolites detected in fermentation broth at different fermentation times at 0, 2, 4, 8, 12, 24, 48 h, respectively.

Name	0h	2h	4h	8h	12h	24h	48h
M0	8.63E+07	3.43E+07	1.89E+07	1.07E+07	2.22E+05	7.28E+04	3.63E+04
M1	2.06E+07	5.25E+07	5.35E+07	4.53E+07	1.76E+05	-	-
M2	1.51E+07	5.31E+07	3.55E+07	2.28E+07	5.98E+05	1.85E+05	1.02E+05
M3	6.46E+07	4.00E+07	3.04E+07	2.00E+07	2.88E+06	-	-
M4	0.00E+00	3.18E+06	8.29E+05	1.40E+06	8.73E+04	-	-
M5	-	1.11E+06	1.17E+06	7.08E+06	-	-	-
M6	-	2.53E+07	1.54E+07	9.38E+06	2.09E+05	-	-
M7	-	5.49E+07	5.32E+07	4.54E+07	3.01E+05	-	-
M8	-	5.07E+08	3.29E+08	3.29E+08	9.20E+06	-	-
M9	-	1.70E+07	1.20E+07	7.35E+06	1.19E+06	9.51E+03	-
M10	-	1.43E+06	1.12E+06	6.53E+05	-	-	-
M11	-	1.43E+06	3.03E+06	1.02E+06	1.08E+05	2.37E+04	3.37E+04
M12	-	1.01E+08	7.40E+07	1.19E+07	-	-	-
M13	-	4.41E+05	5.78E+05	4.68E+02	-	-	-
M14	-	1.50E+05	6.44E+04	-	-	-	-
M15	-	1.23E+05	9.36E+04	5.43E+03	-	-	-
M16	-	6.29E+04	3.62E+05	2.51E+06	5.35E+03	-	-
M17	-	-	5.08E+03	4.85E+03	1.82E+04	6.75E+05	9.95E+07
M18	-	-	4.80E+05	2.62E+06	4.92E+06	6.27E+06	-
M19	-	4.39E+05	3.76E+06	1.21E+07	8.43E+06	8.83E+06	-
M20	-	5.50E+06	2.08E+07	4.25E+07	1.79E+07	1.32E+07	5.85E+04
M21	-	1.20E+05	1.74E+06	1.23E+07	1.64E+07	3.56E+07	9.60E+05
M22	-	3.53E+05	1.80E+06	9.52E+06	1.83E+06	1.93E+06	-
M23	-	-	-	-	-	1.02E+06	1.56E+09
M24	-	-	-	-	9.78E+03	8.52E+05	1.39E+08
M25	-	1.40E+04	1.68E+05	5.33E+02	1.83E+06	2.44E+06	2.98E+05
M26	-	8.84E+04	7.58E+05	3.74E+06	5.82E+06	7.87E+06	7.65E+06
M27	-	2.85E+06	2.68E+06	1.92E+07	1.75E+06	1.17E+06	3.38E+05
M28	-	-	3.75E+04	3.84E+05	1.31E+06	3.89E+06	2.86E+05
M29	7.40E+04	8.29E+04	7.85E+04	9.77E+04	9.84E+04	1.30E+05	2.07E+05
M30	-	-	-	3.31E+05	2.59E+05	1.42E+05	8.12E+04
M31	-	-	-	1.35E+04	1.99E+04	7.50E+04	2.25E+05
M32	2.23E+05	2.89E+06	2.76E+06	3.15E+06	2.36E+06	1.91E+06	-
M33	-	-	-	-	-	1.03E+04	1.42E+06
M34	-	-	-	-	7.33E+04	8.64E+03	2.55E+04
M35	3.43E+06	3.16E+06	3.57E+06	5.94E+06	5.58E+06	5.94E+06	7.31E+06

Table S2 The maximal concentrations of metabolites detected in fermentation broth with the time at which it was measured.

Name	Concentrations (μg/mL)	Fermentation Times
M0	43.23	0h
M1	26.83	4h
M2	26.63	2h
M3	20.08	2h
M4	1.67	2h
M5	3.62	8h
M6	12.73	2h
M7	27.53	2h
M8	253.58	2h
M9	8.58	2h
M10	0.80	2h
M11	1.60	4h
M12	50.58	2h
M13	0.37	4h
M14	0.16	2h
M15	0.15	2h
M16	1.34	8h
M17	49.83	48h
M18	3.22	24h
M19	6.13	8h
M20	21.33	8h
M21	17.88	24h
M22	4.84	8h
M23	780.08	48h
M24	69.58	48h
M25	1.30	24h
M26	4.02	24h
M27	9.68	8h
M28	2.03	24h
M29	0.19	48h
M30	0.25	8h
M31	0.20	48h
M32	1.66	8h
M33	0.79	48h
M34	0.12	12h
M35	3.74	48h

Note: The concentrations of these metabolites were calculated based on the calibration curves of EGCG ($R^2 > 0.99$)

Table S3 Basic information of volunteers

NO.	Gender	Age	BMI
Volunteer 1	Male	21	20.24
Volunteer 2	Male	23	21.37
Volunteer 3	Male	23	22.64
Volunteer 4	Male	24	22.64
Volunteer 5	Female	22	21.30
Volunteer 6	Female	22	19.23
Volunteer 7	Female	23	19.98
Volunteer 8	Female	24	20.20
